# Obesity induced by high-fat diet is associated with critical changes in biological and molecular functions of mesenchymal stromal cells present in visceral adipose tissue

**DOI:** 10.18632/aging.202423

**Published:** 2020-12-27

**Authors:** Mustafa Burak Acar, Şerife Ayaz-Güner, Giovanni Di Bernardo, Hüseyin Güner, Ayşegül Murat, Gianfranco Peluso, Servet Özcan, Umberto Galderisi

**Affiliations:** 1 Genome and Stem Cell Center (GENKÖK) Erciyes University, Kayseri, Turkey; 2 Department of Molecular Biology and Genetics, Faculty of Life and Natural Science, Abdullah Gül University, Kayseri, Turkey; 3 Department of Biology, Faculty of Science, Erciyes University, Kayseri, Turkey; 4 Department of Experimental Medicine, Luigi Vanvitelli Campania University, Naples, Italy; 5 Research Institute on Ecosystems (IRET), CNR, Naples, Italy; 6 Sbarro Institute for Cancer Research and Molecular Medicine, Center for Biotechnology, Temple University, Philadelphia, PA 19122, USA

**Keywords:** mesenchymal stromal cells, visceral adipose tissue, senescence

## Abstract

The mesenchymal stromal cells (MSCs) residing within the stromal component of visceral adipose tissue appear to be greatly affected by obesity, with impairment of their functions and presence of senescence.

To gain further insight into these phenomena, we analyzed the changes in total proteome content and secretome of mouse MSCs after a high-fat diet (HFD) treatment compared to a normal diet (ND). In healthy conditions, MSCs are endowed with functions mainly devoted to vesicle trafficking. These cells have an immunoregulatory role, affecting leukocyte activation and migration, acute inflammation phase response, chemokine signaling, and platelet activities. They also present a robust response to stress. We identified four signaling pathways (TGF-β, VEGFR2, HMGB1, and Leptin) that appear to govern the cells’ functions.

In the obese mice, MSCs showed a change in their functions. The immunoregulation shifted toward pro-inflammatory tasks with the activation of interleukin-1 pathway and of Granzyme A signaling. Moreover, the methionine degradation pathway and the processing of capped intronless pre-mRNAs may be related to the inflammation process.

The signaling pathways we identified in ND MSCs were replaced by MET, WNT, and FGFR2 signal transduction, which may play a role in promoting inflammation, cancer, and aging.

## INTRODUCTION

Worldwide, obesity and its related diseases are among the primary causes of mortality and morbidity. The World Health Organization estimates that more than 1 billion adults are overweight [1]. Obesity is a complex disease that has its roots in excess of food intake, alteration of basal metabolism, and low energy expenditure, although genetic and epigenetic factors also play a role. White adipose tissue (WAT) dysfunction is the primary consequence of obesity; this phenomenon leads to chronic inflammation, as well as cardiovascular and other metabolic pathologies [2].

WAT is present in many depots in our body and can be classified in subcutaneous (sWAT), visceral (vWAT), and bone marrow (bWAT) fat. These WATs possess specific metabolic and inflammatory functions [3, 4]. The unique function of WAT is the regulation of body energy homeostasis by modulating lipid and glucose metabolism. WAT has also a key endocrine role, since adipocytes secrete adipokines that regulate food intake, energy expenditure, reproductive activity, cell death, and inflammation [4].

Alteration of WAT functions induced by obesity also impact stem cells residing in adipose tissue; these cells include those present in mesenchymal stromal cells (MSCs), which are a heterogeneous population consisting of stromal cells, progenitor cells, fibroblasts, and stem cells. The impairment of MSC activities has huge repercussions for health, given the role of MSCs in bone, cartilage, and fat tissue regeneration and in the body’s homeostasis [5].

In a previous study, we demonstrated that obesity induced by a high-fat diet (HFD) profoundly affected the function of MSCs obtained from bone marrow and from visceral and subcutaneous adipose tissues. Specifically, MSCs obtained from vWAT showed impaired proliferation and onset of senescence; this outcome was associated with abrogation of their paracrine functions, such as detoxification activity in response to toxic substances and drugs [6, 7]. This finding is interesting, since vWAT greatly contributes to the negative effects of obesity on human health, as accumulation of visceral fat increases the risk of cardiovascular diseases and type 2 diabetes [8].

These results are in line with other investigations showing that obesity impairs the functions of stem cells residing within WAT by altering the autophagy/mitophagy balance and promoting oxidative and endoplasmic reticulum stress [9–12].

We decided to further investigate the effects of HFD on MSCs residing in vWAT by evaluating how obesity affects the proteome profile of MSCs other than the secretome composition. As a starting point, we considered the presence of senescence phenomena in MSC cultures obtained from obese mice. Senescence is not a static endpoint process: rather, it is a dynamic phenomenon triggered by stress agents that produce genetic and epigenetic changes. The initial phase (early senescence) is associated with a stable cell-cycle arrest that is sustained by P53 and retinoblastoma pathways. Extensive chromatin remodeling and production of a senescence-associated secretory phenotype (SASP) mark the progression to full senescence. The SASP modifies its composition and has increasingly-greater reach in pro-inflammatory factors over time [13, 14]. In this context, we aimed to investigate the late effects of senescence phenomena; therefore, we prolonged the HFD treatment to 20 weeks. This endpoint was carefully selected. After 10 weeks of HFD treatment, we had obese mice with senescent MSCs; extending the HFD regimen to 20 weeks would allow full development of senescence but would also avoid onset of obesity-related pathologies that would complicate the analysis of senescence in MSCs and the evaluation of released SASP [13–16].

## RESULTS

**Figure 1:**
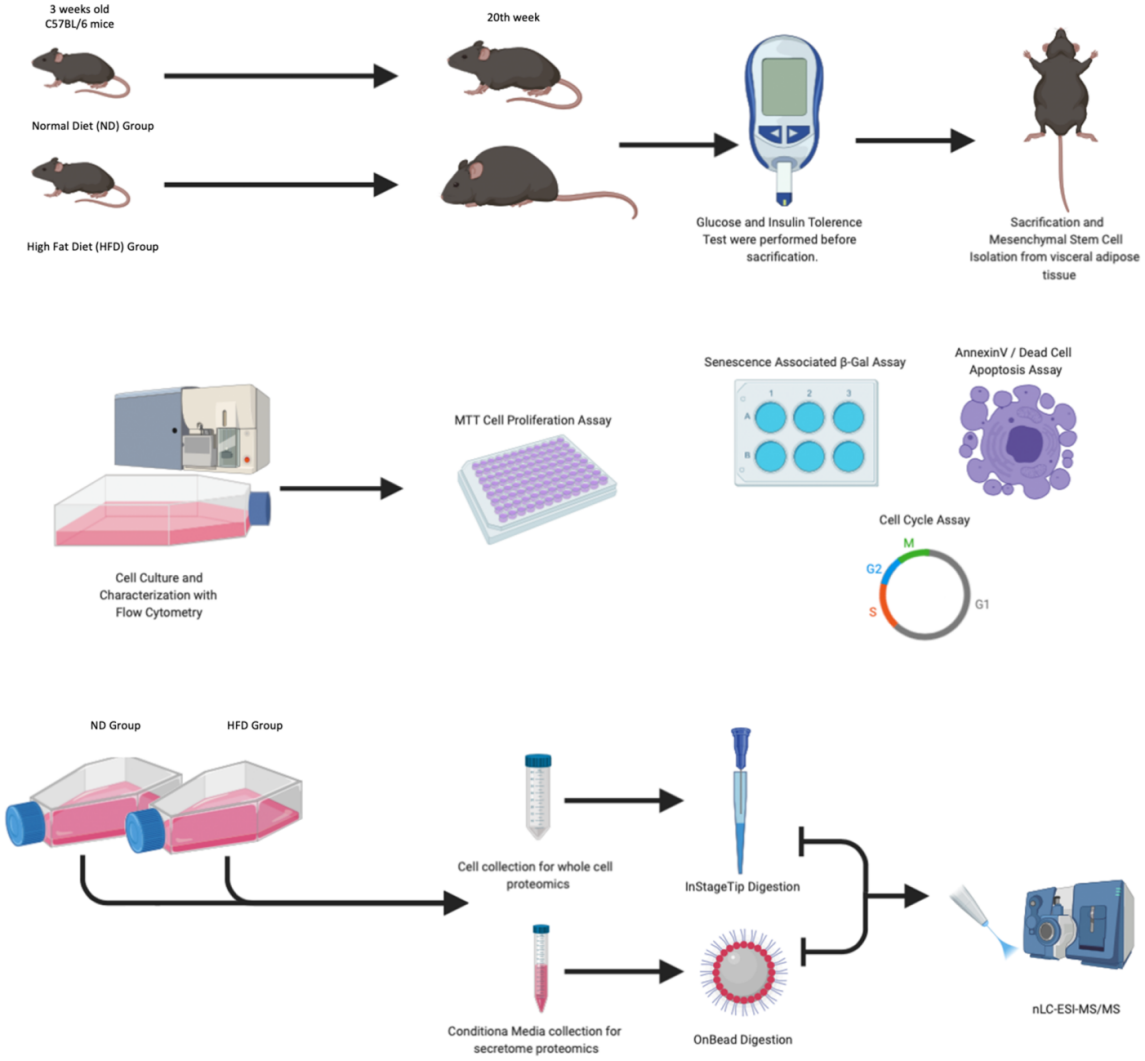
**Experimental design.** Mice were fed with a normal diet (ND) and a high-fat diet (HFD) for 20 weeks. One week before animal euthanasia, GTT and ITT tests were conducted. The MSCs isolated from visceral adipose tissue were characterized by flow cytometry, senescence-associated β-galactosidase assay, annexin V staining, and cell analysis performed at passage 3. At passage 3, for each experimental group, we collected total cellular protein content and secretomes as well as performed LC-MS/MS analysis.

**Figure 2:**
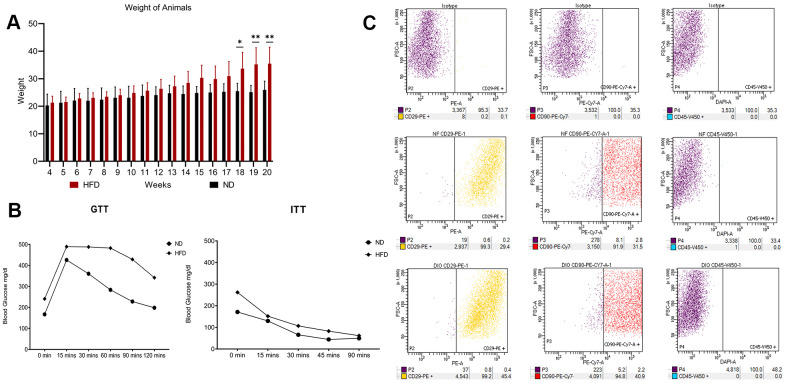
**High fat diet outcomes.** (**A**) Average body weight of animals through 20 weeks. The histogram shows the weight of the five mice fed with HFD and the five mice with ND for 20 weeks. Data are shown with standard deviation (SD) n=5 *p<0.05, **p<0.01. (**B**) Insulin tolerance tests (ITT) and glucose tolerance tests (GTT). The graphs show blood glucose levels (mg/dl) in HFD- and ND-treated mice. (**C**) Flow cytometry results on visceral adipose-derived MSCs. The picture shows a representative example of flow cytometry analysis carried out on P0 MSCs obtained from an ND-treated mouse. Cells were positive for MSC markers CD29 and CD90 and negative for MSC marker CD45. The same results were obtained with cells isolated from HFD-treated mice (data not shown).

The experimental plan of our research is summarized in Figure 1. In the procedure, 3-week-old C57Bl6 mice were fed with a HFD or a standard normal diet (ND) for 20 weeks. At the end of this period, the difference in average body weight between HFD mice and ND mice was approximately 10–12 grams (Figure 2A). Blood glucose levels were measured, and ITT and GTT tests were conducted before the mice were euthanized. HFD mice showed pre-diabetic signs compared to ND mice (Figure 2B).

**Figure 3:**
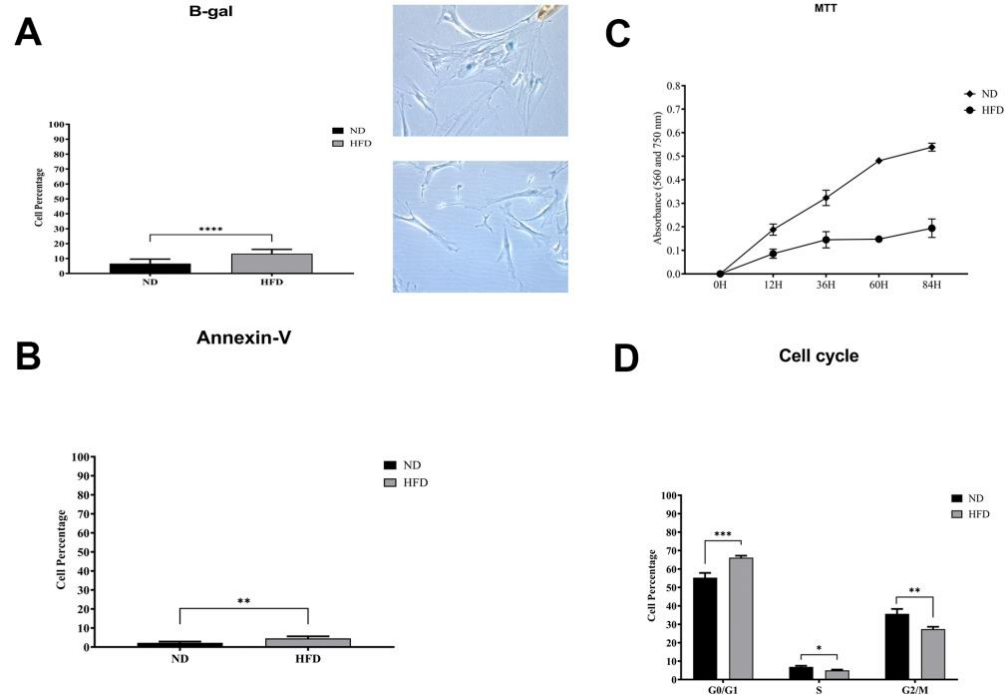
**Biological assays in HFD- and ND-MSCs.** (**A**) The histogram shows the mean percentage value of senescent cells, determined by beta-galactosidase assay. Data are expressed with SD (n = 5) ****p<0.001. Representative images of senescent cells that are stained blue are shown. (**B**) The histogram shows the mean percentage of annexin V-positive cells. Data are expressed with standard deviation (n = 5) **p<0.01. (**C**) Cell proliferation was evaluated by MTT colorimetric assay. The graph shows data coming from HFD and ND samples. (**D**) Representative cell cycle analysis of MSCs harvested from HFD- and ND-treated mice. Data are expressed with SD (n = 5) *p<0.05, **p<0.01, ***p<0.001.

We isolated and cultivated MSCs from the visceral adipose tissue of HFD and ND mice. These cells showed typical mesenchymal markers: CD29(+), CD90(+), and CD45(-) (Figure 2C). As expected, cells from obese mice—hereafter referred to as HFD-MSCs—show higher senescent profiles and lower proliferation rates compared to cells in control samples (hereafter ND-MSCs) (Figure 3A, 3C). This result was in concordance with the reduced percentage of cells in the S and G_2_/M phase and the increase of cells in G_1_/G_0_ (Figure 3D). A slight increase in apoptosis rate was also detected (Figure 3B). This result indicates that a 20-week HFD regimen further exacerbates the senescence phenotype already detected after 10 weeks of treatment. In line with this, we isolated cellular and secreted proteins from MSC cultures to evaluate how obesity affected the protein composition of MSCs.

The LC-MS/MS analyses of peptides from the tryptic digestion of cell lysate samples obtained from HFD- and ND-MSCs identified 2,677 proteins in ND-MSCs and 2,841 in HFD-MSCs; the corresponding secretomes contained 821 and 873 proteins, respectively (Supplementary File 1). The Venn analysis showed that 620 cellular proteins and 122 secreted proteins were exclusively present in ND-MSCs, while 457 cellular proteins and 174 secreted proteins were exclusively present in HFD-MSCs (Supplementary File 1). This result indicates that obesity profoundly affected the cellular protein profiles of MSCs as well as their secretomes. At that point, we performed several bioinformatics analyses to gain insights on differences in protein composition between cells obtained from normal mice and those obtained from obese mice.

We used an integrative approach with complementary bioinformatics tools to develop mechanistic views on changes in protein expression profiles. Gene Ontology (GO) analysis identified the overrepresented proteins (ontology terms) in our ND and HFD datasets (compared to a reference mouse protein dataset). This analysis gives a first impression on which molecular functions and biological activities are involved in cells’ response to environmental changes, such as diet-induced obesity. Then, we integrated the GO classification of proteins present in our samples with pathway analysis and assumed that the most important proteins of a given ontology group are part of the same pathway. At that point, pathway analysis was performed using Reactome software and IPA canonical pathway analysis. The core unit of the Reactome analysis is the reaction: any biologically-active compounds (nucleic acids, proteins, small molecules) participating in reactions form interaction networks that are grouped into pathways. In the IPA analysis, the canonical pathways are well-defined signaling pathways that have been curated by information coming from scientific literature.

### Gene ontology and pathway analyses show differences in the core activities of MSCs obtained from ND and HFD mice

We evaluated the overrepresented ontological terms belonging to the GO classes of biological process (BP) and molecular function (MF). This procedure identified hundreds of ontologies in cell lysates and secretomes obtained from both ND- and HFD-MSCs (Supplementary File 2). We then performed a Venn analysis to identify both the unique and the common ontologies among the cellular and secreted proteomes of MSC samples. The cellular protein analysis revealed 69 BP and 13 MF ontologies specific to ND-MSCs, as well as 30 BP and 4 MF ontologies specific to HFD-MSCs (Supplementary File 2). The evaluation of secreted proteins allowed us to identify 50 BP and 6 MF ontologies specific to ND-MSCs, as well as 40 BP and 11 MF ontologies specific to HFD-MSCs (Supplementary File 2).

Then, we organized the identified specific ontologies into classes of the cell’s core activities to determine the functional effects of obesity on MSCs. We identified eight core activities: cell cycle/cell division, DNA damage, RNA ana-/catabolism, vesicle trafficking, immune system regulation, platelet functions, response to stress, and signaling (Tables 1–4).

**Table 1:** ND-MSC proteome.

	**GO BP**	**GO MF**	**Reactome**	**Canonical pathways**
**Cell cycle/cell division**	Mitotic cell cycle process Mitotic nuclear division Microtubule cytoskeleton organization involved in mitosis	DNA-directed 5'-3' RNA polymerase activity	Amplification of signal from the kinetochores EML4 and NUDC in mitotic spindle formation Resolution of Sister Chromatid Cohesion	
**DNA damage**			Formation of TC-NER Pre-Incision Complex Neddylation	
**RNA ana/catabolism**	Positive regulation of transcription mRNA transport maturation of SSU-rRNA from tricistronic rRNA transcript	RNA polymerase activity		
**Vesicle trafficking** **Eso/endocytosis**	Golgi organization Late endosome to vacuole transport Vesicle targeting, trans-Golgi to periciliary compartment		COPII-mediated vesicle transport	
**Immune system activity**				T Cell Receptor Signaling PI3K Signaling in B Lymphocytes
**Platelet functions**			Platelet degranulation Response to elevated platelet cytosolic Ca2+	
**Response to stress**		NADH dehydrogenase activity		Glutathione Redox Reactions I
**Signaling**	Regulation of cAMP-mediated signaling Receptor signaling pathway via STAT Inositol phosphate signaling Cytokine signaling pathway		Signaling by Rho GTPases RHO GTPases activate CIT RHO GTPases activate KTN1 RHO GTPases Activate ROCKs Signal transduction by L1	NF-kB Signaling VEGF Family Ligand-Receptor Interactions TGF-beta Signaling Galphaq Signaling Leptin Signaling in Obesity HMGB1 Signaling

**Table 2:** HFD-MSC proteome.

	**GO BP**	**GO MF**	**Reactome**	**Canonical pathways**
**Cell cycle/cell division**	Positive regulation of cell population proliferation	DNA-dependent ATPase activity	AURKA Activation by TPX2 Regulation of PLK1 Activity at G2/M Transition	Kinetochore Metaphase Signaling Pathway Cell Cycle: G2/M DNA Damage Checkpoint Regulation Cell Cycle Control of Chromosomal Replication
**DNA damage**	Double-strand break repair via break-induced replication		SUMOylation of DNA damage response and repair proteins Apoptotic execution phase	
**RNA ana/catabolism**	RNA phosphodiester bond hydrolysis, endonucleolytic Regulation of RNA stability Regulation of mRNA catabolic process RNA 3'-end processing Spliceosomal snRNP assembly	snRNA binding	Processing of Capped Intronless Pre-mRNA tRNA Aminoacylation	PRPP Biosynthesis I
**Vesicle trafficking** **Eso/endocytosis**	Golgi to plasma membrane transport Post-Golgi vesicle transport Import into cell		Cargo recognition for clathrin-mediated endocytosis	
**Immune system activity**			Interleukin-1 family signaling	
**Platelet functions**				
**Response to stress**	Response to nitrogen compound Cellular response to organonitrogen compound	Proton transmembrane transporter activity Anion transmembrane transporter activity		
**Signaling**	Regulation of signaling		Intracellular signaling by second messengers MAPK family signaling cascades PIP3 activates AKT signaling Signaling by BRAF and RAF fusions Signaling by FGFR2 Signaling by MET Signaling by WNT	CD40 Signaling 3-phosphoinositide Biosynthesis

**Table 3:** ND-MSC secretome.

	**GO BP**	**GO MF**	**Reactome**	**Canonical pathways**
**Cell cycle/cell division**			Chk1/Chk2(Cds1) mediated inactivation of Cyclin B:Cdk1 complex	
**DNA damage**				
**RNA ana/catabolism**	Positive regulation of transcription RNA metabolic process Regulation of gene expression			
**Vesicle trafficking** **Eso/endocytosis**				
**Immune system activity**	myeloid leukocyte migration leukocyte chemotaxis leukocyte migration Class I MHC mediated antigen processing and presentation		Adaptive Immune System	Natural Killer Cell Signaling Acute Phase Response Signaling Chemokine Signaling Complement System
**Platelet functions**			Hemostasis	
**Response to stress**	Response to endoplasmic reticulum stress	Disulfide oxidoreductase activity	Detoxification of Reactive Oxygen Species	
**Signaling**	Enzyme linked receptor protein signaling pathway	Growth factor binding		PAK Signaling CXCR4 Signaling Phospholipase C Signaling LXR/RXR Activation Aryl Hydrocarbon Receptor Signaling

**Table 4:** HFD MSC secretome.

	**GO BP**	**GO MF**	**Reactome**	**Canonical pathways**
**Cell cycle/cell division**			Loss of Nlp from mitotic centrosomes Regulation of PLK1 Activity at G2/M Transition	
**DNA damage**				
**RNA ana/catabolism**	Regulation of transcription initiation from RNA polymerase II promoter Regulation of mRNA processing mRNA metabolic process RNA processing		Processing of Capped Intron-Containing Pre-mRNA mRNA Splicing - Major Pathway	Spliceosomal Cycle
**Vesicle trafficking** **Eso/endocytosis**	Intra-Golgi vesicle-mediated transport		Membrane Trafficking Vesicle-mediated transport Transport to the Golgi ER to Golgi Anterograde Transport COPI-dependent Golgi-to-ER retrograde traffic	
**Immune system activity**	Regulation of immune system process Innate immune response Immune effector process Positive regulation of lymphocyte activation			Granzyme A Signaling Superpathway of Methionine Degradation
**Platelet functions**				
**Response to stress**		Heat shock protein binding	HSF1 activation Regulation of HSF1-mediated heat shock response Chaperone Mediated Autophagy Cellular response to heat stress Defective B4GALT7 causes progeroid syndr.	Superoxide Radicals Degradation Oxidized GTP and dGTP Detoxification
**Signaling**	Regulation of transmembrane receptor protein Ser/thr kinase signaling pathway	Protein kinase activity	Signaling by PDGF Signaling by MET	Sirtuin Signaling Pathway

Reactome analysis identified 258 pathways in the proteomes of ND-MSCs and 256 in the proteomes of HFD-MSCs. The pathways in the corresponding secretomes were 230 and 264, respectively. We then performed Venn analysis to determine the cell type-specific pathways; we identified 19 pathways exclusively present in proteomes of ND-MSCs and 17 pathways exclusively present in proteomes of HFD-MSCs. The corresponding secretomes contained 8 and 32 exclusive pathways, respectively (Supplementary File 3). These specific pathways were also classified according to the core activities indicated above (Tables 1–4).

We then analyzed the canonical pathways with IPA software, which identified 292 pathways in the proteomes of ND-MSCs and 281 in the proteomes of HFD-MSCs. The numbers of canonical pathways in the corresponding secretomes were 230 and 264, respectively. Venn analysis determined the cell type-specific pathways: 23 pathways were exclusively present in the proteomes of ND-MSCs, and 12 were exclusively present in those of HFD-MSCs. The corresponding secretomes showed 20 and 9 exclusive pathways, respectively (Supplementary File 3). The pathways were classified according to the identified core activities (Tables 1–4).

### MSC cellular protein content is profoundly modified by obesity

Both ND and HFD proteomes are enriched in the specific signaling that is related to the G_2_/M phase and mitosis, such as mitotic assembly checkpoint and the Aurora kinase and PLK1 pathways for ND samples (Tables 1, 2). The DNA damage task in ND samples appears to be more focused on single-strand DNA repair, given the presence of specific pathways associated with transcription coupled-nucleotide excision repair (TC-NER). The TC-NER is the most versatile and important DNA repair mechanism that allows cells to cope with daily DNA damage events that may occur during a cell’s lifetime [17]. Ubiquitin and the ubiquitin-like proteins (UBL) SUMO and NEDD8 play a role in regulating the cellular response to DNA damage [18]. The ND cellular proteome is endowed with proteins involved in neddylation (NEDD8) pathways. In contrast, the HFD samples are endowed with proteins involved in DNA double-strand break repair—such as SUMO ligases, which add SUMO peptides to BRCA1, HERC1, RNF168, MDC1, and TP53BP1 [19]. This result may indicate that HFD-MSCs have to cope with stronger genotoxic injuries compared to ND-MSCs. Indeed, obesity—with its related increases in reactive oxygen species (ROS) and in pro-inflammatory factors—may promote DNA damage events.

Both ND and HFD proteomes are enriched in proteins involved in RNA metabolism; ND samples are biased toward anabolic processes, such as positive regulation of RNA biosynthetic processes and of transcription, while HFD samples contain more factors involved in RNA catabolism (RNA phosphodiester bond hydrolysis, endonucleolytic regulation of RNA stability). Notably, HFD proteomes are endowed with proteins involved in the processing of capped intronless pre-mRNAs (Table 2). The intronless genes (IGs) are a small proportion of all genes, and the majority of IGs encode for G-coupled receptors and for other proteins with signal transduction activity. Another big IG group encodes for histones and chromatin-remodeling proteins. In addition, some IGs encode for genes involved in inflammation (15 interferon encoding genes) [20]. Most of the proteins encoded by IGs may be part of the pathways and ontological groups identified in the HFD samples, such as those regulating inflammation and signaling (see below).

MSCs have great vesicle trafficking, since they release factors that are synthesized on the endoplasmic reticulum and go through the Golgi apparatus to the exocytotic route; moreover, they play a role in MHC I antigen processing [21]. In this context, it is not surprising that both ND and HFD proteomes are enriched in proteins that regulate Golgi organization, Golgi-to-plasma membrane transport, cargo recognition, and clathrin-mediated endocytosis (Tables 1, 2).

MSCs play a role in immune system regulation and hemostasis [5, 22]. Indeed, ND proteomes specifically contain proteins involved in platelet ligand-dependent activation and the ensuing degranulation with the release of platelet agonists, glycosidases, acid proteases, adhesive proteins, prothrombotic factors, and pro-inflammatory factors [6, 7]. ND proteomes are also endowed with proteins involved in T cell receptor signaling and in PI3K signaling in B lymphocytes. In contrast, HD proteomes do not contain proteins involved in platelet functions, and the proteins involved in immune regulation are primarily associated with pro-inflammatory pathways (e.g., Interleukin-1 family signaling) (Tables 1, 2) [23].

Cellular response to stress is different in ND and HFD samples. In the latter proteome, we identified ontologies and pathways generally associated with cellular response to organonitrogen compounds. This class of chemical compounds contains numerous diverse molecules that may have a role in either cell physiological functions or cell stress. ND proteomes, in contrast, are enriched in factors that can actively cope with endogenous and external reactive oxygen stress, such as pathways related to NADH dehydrogenase and glutathione redox activity [24].

**Figure 4:**
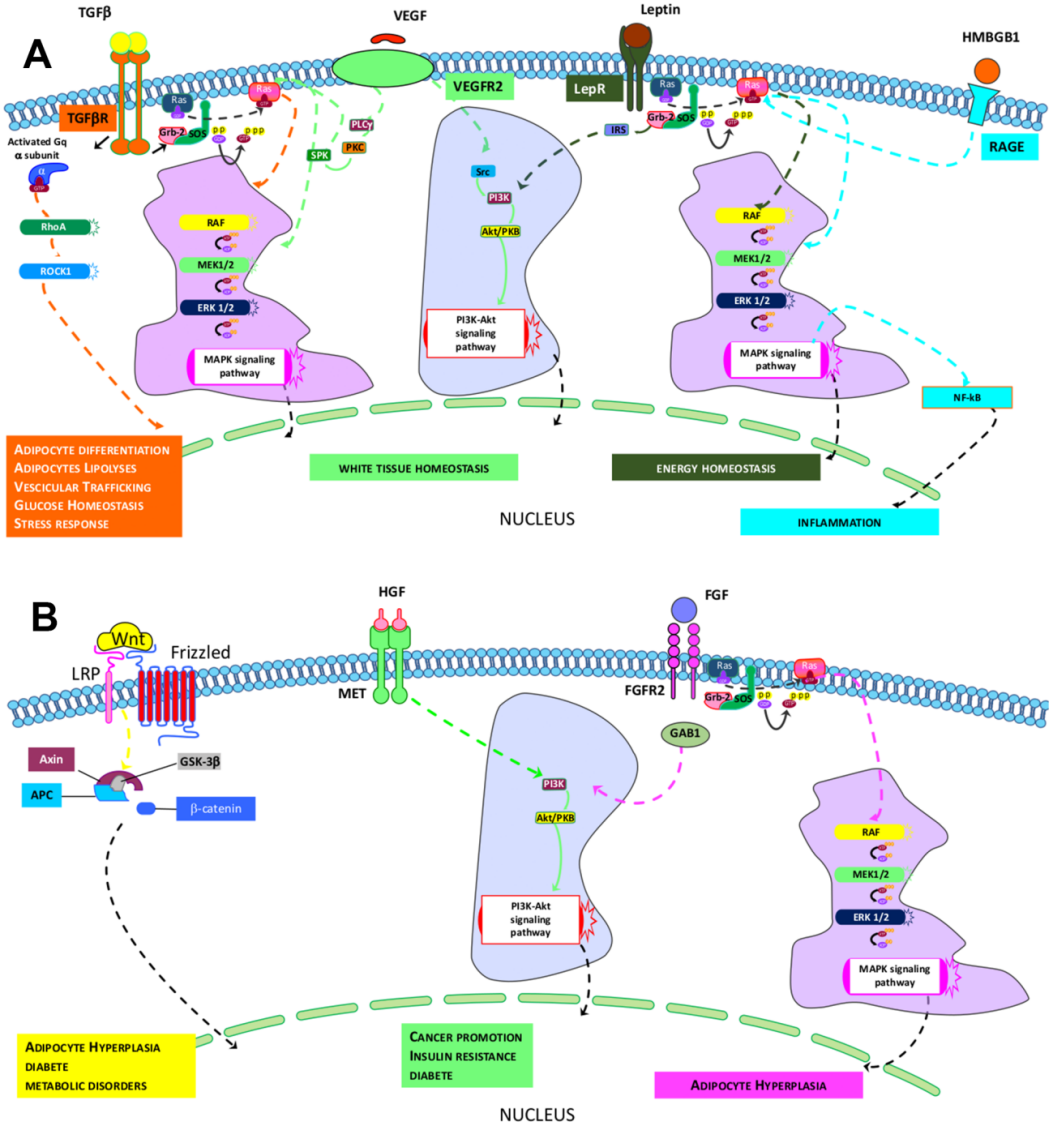
**Identified pathways in ND- and HFD-MSCs.** (**A**, **B**) The pictures show the signaling pathways that govern the specific core biological activities (see Table 1) of ND-MSCs and HFD-MSCs, respectively.

In terms of the key signaling circuitries that govern the above-indicated activities, a comparison of Reactome and canonical pathway analyses allowed us to identify transduction signal pathways in cells under investigation. The ND-MSCs rely on signaling coming from TGF-β, VEGFR2, HMGB1, and Leptin pathways, while the HFD-MSCs depend on MET, WNT, and FGFR2 signal transduction (Figure 4A, 4B). The four paths of ND-MSCs rely on RAS/MAPK downstream signaling. The TGF-β path also requires Gαq/RHO/RHOCK signaling, while the others may work through PI3K/AKT signals. Finally, HMGB1 and leptins may also have a connection with the NF-κB path (Figure 4A).

The MET and FGFR2 pathways identified in HFD-MSCs act downstream via PI3K/AKT, while WNT appears to work through canonical β-catenin signaling (Figure 4B).

### Indications of regulators that control expression of proteins present in HFD and ND samples

LC/MS-MS analysis of cell proteomes may not allow the identification of all proteins present in a cell lysate, since cellular proteins range widely and dynamically in their concentrations. This enables the signal from the most abundant proteins to mask the signal of less-abundant ones, such as transcription factors and other regulatory molecules. We used the IPA Causal Network Analysis (CNA) to overcome this methodological limit. The CNA allows us to infer upstream molecules that control the expression of the genes/proteins in a given dataset.

We focused our interest on transcription factors that can be involved in the regulation of the genes that encode the proteins present in our proteome samples. The Venn analysis identified 70 upstream regulators exclusively present in ND-MSCs and 33 exclusively present in HFD-MSCs (Supplementary File 3). Among these regulators, we identified 6 transcription factors (GLI1, NR1I2, NR1I3, SMAD4, KLF6, and ARNT2) that can control the expression of proteins present in ND-MSCs, and we identified 2 (RARA and NKX2-3) that appear to be involved in gene expression regulation in HFD-MSCs.

### Obesity-affected secretome composition of MSCs

In healthy conditions, MSCs secrete factors that are related to the specific cellular functions (core activities) we identified in our analysis of the cellular proteome content. ND-MSCs released proteins involved in immune system regulation and platelet activities, such as leukocyte migration and chemotaxis, natural killer cell signaling, chemokine signaling, class I MHC mediated antigen processing/presentation, and hemostasis. ND-MSCs also secreted proteins involved in cell response to stress, such as enzymes with disulfide oxidoreductase activity (detoxification of ROS and response to endoplasmic reticulum stress) (Table 3 and Supplementary Files 2, 3). The secreted proteins of ND-MSCs are enriched in signaling pathways that express MSC physiological functions, such as PAK signaling, CXCR4 signaling, LXR/RXR activation, and aryl hydrocarbon receptor signaling. The P21-activated kinases (PAKs) are serine/threonine protein kinases involved in pathways that play a role in migration, proliferation, apoptosis, mitosis, and vesicle-mediated transport processes [25]. The CXCR4 surface chemokine receptor pathway is involved in the mobilization of MSCs [26]. The activation of the LXR/RXR pathway improves glucose tolerance through coordinated regulation of glucose metabolism in liver and adipose tissues [27]. The aryl hydrocarbon receptor signaling originally functions primarily as a sensor of toxic substances and as a regulator of enzymes involved in stress relief and detoxification [28].

Several changes were seen in the secretome composition of HFD-MSCs. We found factors involved in immune system regulation, such as positive regulation of lymphocytes activation, granzyme A signaling, and methionine degradation. The stress response factors released by HFD-MSCs were those that are involved in regulating heath shock response, chaperone-mediated autophagy, and superoxide radical degradation. The secretomes of HFD-MSCs contain factors involved in PDGF signaling that, in addition to their mitogenic activity, may regulate local inflammation and promote atherosclerosis [29]. Enrichment in MET signaling found in the proteomes of HFD-MSCs was also detected in their secretomes, and this underscore the key importance of such a network for their functions. Two signaling paths identified in the secretomes of HFD-MSCs were related to senescence and aging phenomena: the sirtuin signaling pathway, and the defective B4GALT7 pathway associated with progeroid syndromes [30, 31].

### Quantitative changes in the protein expression of MSCs following HFD treatment further confirm that obesity promotes senescence and impairment in physiological functions

**Figure 5:**
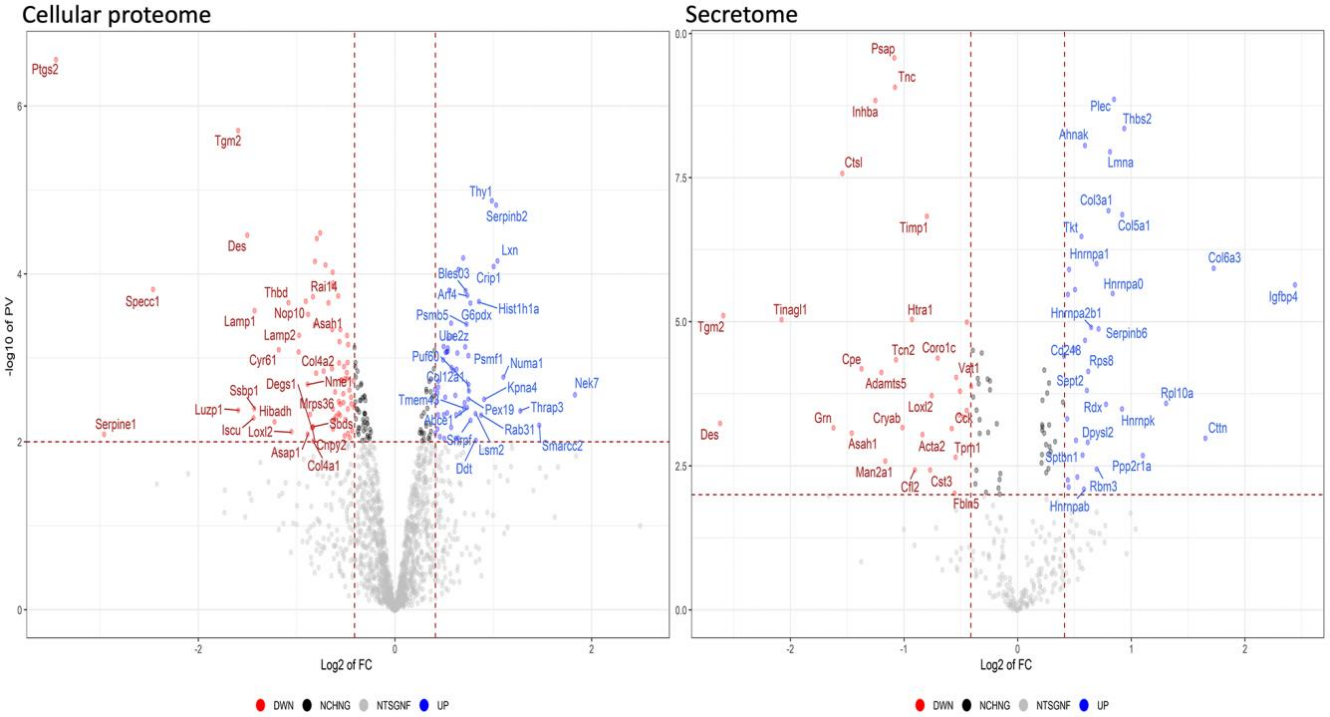
**Quantitative changes in the protein expression of MSCs following HFD treatment.** The volcano plot displays the results of up- and down-regulated proteins in HFD samples compared to ND samples. Panels data from cellular proteome and secretome, respectively.

**Figure 6:**
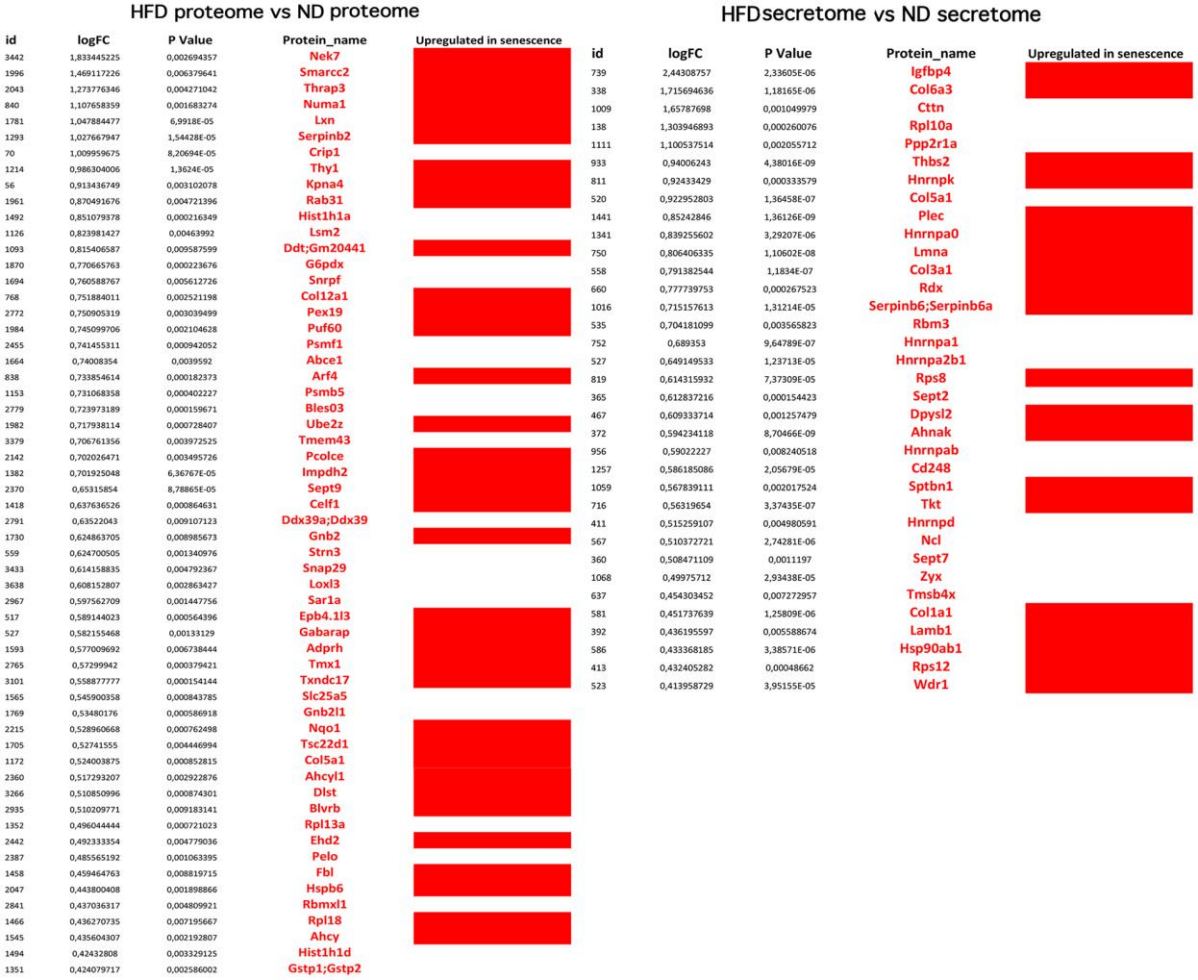
**Overexpressed proteins in HFD-MSCs associated with senescence.** The picture shows the overexpressed (in red) and downregulated (in black) proteins in the proteomes and secretomes of HFD-MSCs compared to ND-MSCs. The differences in expression are expressed as LogFC. The column “Upregulated in senescence” indicates the proteins for which expression is upregulated in senescent cells, according to SeneQuest (https://senequest.net) and PubMed (https://pubmed.ncbi.nlm.nih.gov).

The analyses described thus far focused on identifying biological processes, molecular functions, and related pathways that were specific to either health or pathological conditions of the MSCs. These analyses were conducted considering only the presence or absence of each protein in a given dataset (as compared to reference mouse protein databases). Further insights were obtained by evaluating changes in the expression level of each protein, by means of quantitative proteome analysis. We compared the expression levels of cellular proteins of HFD-MSCs with those of ND-MSCs and considered only the differences in values that were highly significant (*p* < 0.001). We identified 61 upregulated and 74 downregulated proteins in the proteomes of HFD-MSCs and 35 upregulated and 31 downregulated proteins in their secretomes (Supplementary File 4 and Figure 5). We then evaluated how many of these upregulated proteins were related to senescence, using SeneQuest (https://senequest.net) software and literature data to discover proteins associated with senescence. We found that 37 of the 61 upregulated cellular proteins in HFD-MSCs were related to senescence and that 20 of the 35 upregulated secreted factors belong to pathways associated with senescence phenomena (Figure 6) [32, 33].

These results corroborate the finding that obesity promotes senescence of MSCs through production of SASP factors.

## DISCUSSION

Obesity is associated with impairment of adipose tissue’s physiological role and chronic inflammation, which in turn contribute to the onset of several diseases. The MSCs residing in the stromal component of adipose tissue are also affected by negative cues originating from obesity. This impairs organismal homeostasis, since MSCs play a key role in the functionality of bone, cartilage, and fat tissue. The MSCs present in visceral adipose tissue appear to be greatly affected by obesity, with impaired functions and the presence of senescence phenomena [6].

The presence of senescent cells in vWAT is compelling, since it suggests that many pathological effects related to the increase in number and size of adipocytes in this tissue may derive from the presence of senescent cells that actively produce pro-inflammatory, pro-aging and even carcinogenic factors. We decided to further investigate senescence phenomena in the vWAT of mice treated with a HFD. Senescence is complex, since there are different types of senescent cells with specific properties under a common umbrella of shared features. In addition, senescence is a progressive process that begins with a pre-senescent status and eventually reaches full senescence [14]. This final status is the most dangerous for health, since the SASP is endowed with pro-inflammatory cytokines, and the anti-tumor factors present in the SASP of early senescent cells are replaced with factors that can sustain tumor growth [34]. To learn more, we analyzed cellular proteome content and secretome of vWAT-resident MSCs after 20 weeks of HFD treatment to determine a full senescent phenotype.

We performed a robust analysis with bioinformatics methods to thoroughly study the mechanisms behind the effects of obesity on MSC functions. The GO analysis and pathway analysis by Reactome and IPA software allowed us to identify some core activities of MSCs in healthy conditions (Tables 1, 3). In particular, these MSCs are endowed with biological functions and molecular processes, primarily devoted to vesicle trafficking to and from the extracellular environment. These cells have an immunoregulatory role, with effects on leukocyte activation and migration, acute inflammation phase response, chemokine signaling, and platelet activities. They also present a robust response to stress through ROS detoxification.

Our study identified four signaling pathways—TGF-β, VEGFR2, HMGB1, and Leptin—that appear to govern the cells’ functions. The TGF-β pathway appears to act via Gαq/RHO/ROCK and MAPK signaling. Several previous findings have shown that this pathway regulates several aspects of adipose tissue activities. For example, TGF-β signaling is involved in the increase of adipocyte precursor proliferation, thereby promoting hyperplastic vWAT expandability [35]. RHO signaling plays a role in adipogenesis regulation, in glucose homeostasis (by controlling vesicular trafficking), and in adipocyte lipolysis [36, 37]. In addition, Gαq signaling may promote white adipogenesis and inhibit the formation of brown and beige adipose tissue [38]. TGF-β signaling may also play a role in controlling the MSC stress response, since it can induce glutathione peroxidase-1 and prevents ROS-induced cell death [39]. VEGF signaling is crucial for healthy vWAT, since its inactivation may induce WAT expansion, whitening of brown adipose, fat accumulation, and a decrease in energy consumption [40]. The Leptin pathway also plays an essential role in energy homeostasis by stimulating glucose and fatty acid oxidation [41]. Moreover, HMGB1, through the NF-κB path, may regulate the role of MSCs in inflammation [42].

The IPA upstream regulator algorithm identified 6 transcription factors—GLI1, NR1I2, NR1I3, SMAD4, KLF6, and ARNT2—that may govern the expression of proteins identified in the proteomes of ND-MSCs. Three of them (GLI1, SMAD4, and KLF6) are involved in MSC adipogenesis by controlling cell commitment and differentiation [43–45]; NR1I2 and NR1I3 regulate the expression of genes involved in detox activities [46, 47]; and hydrocarbon receptor nuclear translocator 2 (ARNT2) seems to play a role in regulating obesity [48].

In obese mice, the MSCs obtained from vWAT showed a significant change in their biomolecular functions. The GO and pathway analyses revealed that vesicle trafficking from/to the extracellular environment and immunoregulatory activities were present, as observed with ND-MSCs. However, the immunoregulation appears to be shifted toward pro-inflammatory tasks, since we detected activation of interleukin 1 signaling and the implementation of granzyme A signaling, which is related to the activities of Cytolytic T lymphocytes (CTL) and natural killer (NK) cells [49]. Moreover, the methionine degradation pathway [50] and the processing of capped intronless pre-mRNAs [20] may be related to inflammation processes.

We identified several stress response activities (activation of heath shock proteins, chaperone mediated autophagy, and superoxide radical degradation), but these signals seemed insufficient to cope with negative obesity-related cues coming from the extracellular environment. Indeed, the HFD-MSCs showed impaired physiological tasks and the onset of frank senescence phenotypes that promote inflammation and can favor cancer onset and aging phenomena. The signaling pathways we identified in ND-MSCs were replaced by MET, WNT, and FGFR2 signal transduction in HFD-MSCs, which may play a role in the aforementioned pathological conditions. MET signaling may promote cancer recurrence as well as insulin resistance and diabetes [51, 52]; the WNT pathway could be associated with adipocyte hyperplasia, diabetes, and metabolic disorders [53]; and FGFR2 transduction signaling also may play a role in obesity, adipocyte hyperplasia, and cancer [54, 55]. The two upstream regulators—RARA and NKX2-3—appear to play a key role in controlling gene expression in HFD-MSCs and are important for adipocyte metabolism and differentiation; their dysregulation can contribute to metabolic syndrome and inflammation [56–58].

The analysis of overexpressed proteins in HFD-MSCs and of those present in their secretomes further revealed that obesity promotes the onset of full senescence phenomena. Indeed, 37 of 61 upregulated cellular proteins and 20 of 35 upregulated secreted factors belong to pathways associated with senescence.

## CONCLUSIONS

In this study, we analyzed the changes induced by obesity in the cellular and secreted protein profiles of MSCs present in vWAT. We identified the key signaling pathways that are active in healthy conditions and those that induce the phenotypic changes occurring in obesity. Our findings may pave the way for developing therapeutic strategies to fight obesity, either by actively maintaining the signaling circuitries present in healthy conditions or by blocking those that induce senescent and inflammatory phenotypes of MSCs.

## MATERIALS AND METHODS

### Animal work and diet-induced obesity formation

All experiments in this study were carried out in compliance with the approval of Erciyes University’s Animal Experiments Ethical Committee (14^th^ December 2016, 16/164). The animals were obtained from Erciyes University GENKÖK, the transgenic animal department. We fed five C57BL6 mice (3 weeks old) with a Normal Diet (ND) and five other C57BL6 mice with a High-Fat Diet (HFD) for 20 weeks. In the HFD, the pellets contained 60% fat (Custom Diet, Safe, Inserm, France), while the ND pellets contained 5.7% fat. After 20 weeks, when the average weight difference was at least 10 grams, the mice were euthanized.

### Insulin (ITT) and glucose (GTT) tolerance tests

Insulin and glucose tolerance tests were performed before the mice were euthanized. For the GTT, the mice were food-deprived for 12 hours before the test; then, 0.2 g/mL glucose were injected intraperitoneally, and values were measured with Accu-Chek Performa nano (Roche, Switzerland) at 6 time-points (0, 15, 30, 60, 90, and 120 min post-injection). The same food deprivation strategy was used to perform the ITT, whereafter a 0.8 mU/μL insulin solution was injected intraperitoneally and the blood glucose level was measured at five time points (0, 15, 30, 60, and 90 min post-injection).

### Isolation and characterization of visceral adipose MSCs

Animals were euthanized by cervical dislocation. The visceral adipose tissue was isolated and cut into small pieces with surgical blades. Then, tissue samples were incubated for 15 minutes at 37° C in a solution containing 2.5 mg/ml type II collagenase (Sigma-Aldrich, MO, USA). Samples were filtered on cell strainers (70 μm mesh), centrifuged, and washed three times with phosphate buffer saline (PBS). Cells were then plated onto 75-mm flasks with alpha-MEM containing 15% fetal bovine serum (FBS) and were incubated for 7 days to reach confluence (P0). The cells were then trypsinized and characterized by flow cytometry analysis, which was performed with BD FACSAriaIII and BD FACSDiva 8.0.1. software (BD Bioscience, CA, USA). We used CD90 as a positive marker and CD29 and CD45 as negative markers. After P0, cells were cultivated for another 15 days (Passage 3 - 4) for all the biological and proteomic assays.

### Cell cycle analysis

For each analysis, 5x10^4^ cells were collected by trypsin treatment and then, after PBS washing, were fixed in 70% ethanol overnight at -20° C. The samples were then washed with PBS and subsequently dissolved in a hypotonic buffer containing propidium iodide (Millipore, MA, USA). Analyses were performed using Muse Cell Analyzer (Millipore, MA, USA) following the manufacturer’s instructions.

### Senescence-associated β-galactosidase assay

Cells grown in 6 well plates were fixed using a 0.2% glutaraldehyde solution for 5 min at room temperature (RT). Then, the cells were washed with PBS and stained with 40mg/mL X-gal staining solution, as described in [59]. Blue-stained cells were counted from 3 different regions of each well, and the percentage of senescent cells was determined. In identifying senescent cells, we also considered other properties, such as cell size, multi-nuclei presence, and granularity.

### Annexin V and dead cell assay

Apoptosis was detected using the Annexin V and Dead Cell kits (Millipore, MA, USA) on a Muse Cell Analyzer (Millipore, MA, USA) following the manufacturer’s instructions.

### Cell proliferation assay

Cell proliferation was determined by MTT 3-(4,5-dimethylthiazol-2-yl)-2,5-diphenyltetrazolium bromide (MTT) assay. First, 3,000 cells were seeded in each well of 96 well plates. At 12, 36, 60, and 84 hours, we added 1 mg/mL of MTT reagent to the cell media. After 4 hours of incubation at 37° C, the media were removed, and 100 μL DMSO was added to solubilize the salt crystals. The absorbance values were then measured at 560/750 nm using Glomax MultiWP Reader (Promega, WI, USA).

### Whole cell sample preparation for mass spectroscopy

Samples were prepared with the InStage Tip digestion method described by Kulak and colleagues [60]. We collected 1x10^6^ cells each from the NF and HFD groups. After PBS washing, cells were stored at -80° C until lysis step. Then, 100 μL Lysis Buffer (6M Guanidinium chloride, 40mM CAA, 10mM TCEP, 25mM Tris-HCl pH:8,5) was added onto cell pellets and vortexed; subsequently, lysates were boiled for 5 minutes and then incubated on ice for another 5 minutes. After the cold incubation step, samples were sonicated in an ice-filled ultrasonic water bath for 5 minutes. Samples were then centrifuged at 20,000 g for 15 minutes, and proteins containing supernatants were collected.

We mixed 20 μL of each supernatant with 280 ng Lys-C (Promega, WI, USA) containing a 40 μL dilution buffer (25 mM Tris-HCl pH 8.5, % 10 ACN), which we put into InStage tips previously prepared by using 3 SDB-RPS extraction disks (3M Emporem, MN, USA). Mixtures were incubated overnight at 37° C. Subsequently, 1,000 ng Trypsin-Gold (Promega, WI, USA) was added to the Stage Tips, mixed well, and incubated for 4 hours. Following the incubation step, a 140 μL loading buffer (1% TFA) was added to each tip and centrifuged at 2,000 g; then, the peptide-loaded disks were washed four times with a 100 μL washing buffer. Peptides were eluted from disks in three fractions, according to their hydrophobic properties, by using 60 μL of each of the three elution buffers: SDB-RPS1 (100 mM Ammonium formate, 35% ACN, 0.5% Formic Acid), SDB-RPS2 (100 mM Ammonium formate, 55% ACN, 0.5% Formic Acid), and Buffer X (80% ACN, 0.125% Ammonia). Samples were lyophilized with SpeedVac and stored at -20° C until the LC-MS/MS analysis.

### Secretome sample preparation for mass spectroscopy

MSC cultures were incubated in serum-free media for 24 hours; then, 5 mL of culture medium (secretome) was collected from each culture dish without disturbing the attached cells. Culture debris was removed by centrifugation at 10,000 g for 10 minutes, and supernatants were used for the StartaClean beads protein pooling. Collected secretomes were incubated overnight with the beads; then, the beads were washed twice with TE Buffer (50 mM Tris 10 mM EDTA pH 7) and dried with a vacuum concentrator.

The dried beads were resuspended at 2% (w/v) in a RapiGest (Agilent, CA, USA) solution containing TEAB (Sigma, MO, USA). Then, TCEP (Sigma, MO, USA) was added to the solution at a final concentration of 20 mM. Samples were incubated at 60° C for 30 minutes and cooled on ice. IAA (Bio Rad, CA, USA) was added to sample solutions, and samples were incubated at RT for 15 min. Then, 200 ng Lys-C (Promega, WI, USA) was added to each sample and incubated for 4 hours at 37° C. After Lys-C incubation, 800 ng Trypsin-Gold (Promega, WI, USA) was added to each sample and incubated overnight. Samples were centrifuged at 10,000 g for 1 minute; peptides containing supernatants were collected and acidified with 1% TFA before being loaded into Stage Tips. These tips were prepared with C18 material: they were washed with buffer B (% 0.1 Acetic Acid, 80% ACN) and equilibrated with buffer A (% 0.1 Acetic Acid). Acidified samples were loaded onto Stage Tips, and peptide-bounded tips were washed twice with buffer A. Following the washing, buffer B was added to the tips, and samples were eluted into collecting tubes with a syringe. Samples were dried with a vacuum concentrator and stored at -20° C until LC/MS analysis.

### LC-MS/MS analysis

LC-MS analysis was performed with AB Sciex Triple ToF 5600+ (AB SCIEX, CA, USA) integrated with LC-MS/MS Eksigent ekspert™ nanoLC 400 System (AB SCIEX, CA, USA). Peptides were separated using nanoACQUITY UPLC 1,8 μM HSS T3 C18 column (Thermo Fisher, MS, USA) in the trap-elute mode. In order to separate the peptides, 4–40% ACN gradient was used for 240 minutes. Data dependent acquisition (DDA) MS/MS analysis of separated peptides was performed after electrospray ionization. Raw data analysis—generated by instrument reporting—and multiple analytical data measurements in each sample were performed with Analyst® TF v.1.6 (AB SCIEX, CA, USA). The peptides and the ion-product of the MS and MS/MS data were evaluated with PeakView (AB SCIEX, CA, USA). Generated peak-lists were evaluated in consideration of the UniProtKB-based reference library of the *Mus musculus* species on our server with ProteinPilot 4.5 Beta (AB SCIEX, CA, USA).

### Label-free quantification

Quantitative analysis of data was completed by using MaxQuant (1.16.14.0) (https://www.maxquant.org). MS mass tolerance determined as 0.5 Da, MS/MS mass tolerance was 20 ppm. Carbamidomethyl was determined as fixed modification, oxidation and acetylation (protein N-term) were determined as variable modifications. Quantitative changes between groups were analyzed by using Limma, which is an R/Bioconductor software package (https://bioconductor.org/packages/release/bioc/html/limma.html). Visualization of volcano plots was performed with R (R 4.0.2) a software of Limma.

### Bioinformatic evaluation

The cellular proteins and secreted factors identified by LC/MS were analyzed with PANTHER (http://www.pantherdb.org), Reactome Knowledgebase (https://reactome.org), and Ingenuity Pathway Analysis (IPA) (http://www.ingenuity.com/products/ipa). The significance ratio was *p* < 0.01 for all bioinformatic evaluations.

With PANTHER, we performed a Gene Ontology (GO) analysis according to two ontological terms: biological processes and molecular functions. In this analysis, we used statistics overrepresentation to compare classifications of multiple clusters of lists to a reference list in order to statistically identify the over- or under-representation of PANTHER ontologies. We followed the developers’ instructions for running a PANTHER analysis [61].

For the Reactome analysis, the proteins identified by LC/MS were mapped to specific pathways. Over-representation and pathway-topology analyses were conducted. Over-representation analysis is based on statistical hypergeometric distribution; it evaluates whether certain specific Reactome pathways are enriched in the submitted data. This analysis produced a probability score, wherein false discovery rate (FDR) was corrected for using the Benjamani-Hochberg method. We followed the developers’ instructions for running a Reactome analysis [62, 63].

The identified proteins in the different experimental conditions were also imported into IPA to attribute them to canonical pathways. Fischer's exact test was used to calculate a *p*-value that would determine the probability that the association between genes in the dataset and the canonical pathway could be explained by chance alone.

### Biostatistics

Biostatistical analyses were conducted using Graphpad Prism software. Two-way ANOVA was used for analyses of cell cycles, and unpaired *t*-tests were used for the data from other biological assays.

## Supplementary Material

Supplementary File 1

Supplementary File 2

Supplementary File 3

Supplementary File 4
