# Supplementary material for: Obesity induced by high-fat diet is associated with critical changes in biological and molecular functions of mesenchymal stromal cells present in visceral adipose tissue
Source: Aging (Albany NY). 2020 Dec 27;12(24):24894–913. doi: 10.18632/aging.202423 (PMC7803587; doi:10.18632/aging.202423)
Supplement: Supplementary File 2 [file aging-12-202423-s002.pdf]

Supplementary File 2. Gene Ontology and Venn analysis.

Names	total	elements
HFD BP ND BP	417	cellular amino acid biosynthetic process (GO:0008652)
		anterograde trans-synaptic signaling (GO:0098916)
		immune system process (GO:0002376)
		organelle membrane fusion (GO:0090174)
		protein import into nucleus (GO:0006606)
		regulation of anatomical structure size (GO:0090066)
		nucleotide-sugar metabolic process (GO:0009225)
		organic acid biosynthetic process (GO:0016053)
		nuclear transport (GO:0051169)
		leukocyte mediated immunity (GO:0002443)
		regulation of actin polymerization or depolymerization (GO:0008064)
		carbohydrate derivative biosynthetic process (GO:1901137)
		regulation of actin filament polymerization (GO:0030833)
		regulation of microtubule cytoskeleton organization (GO:0070507)
		cellular protein metabolic process (GO:0044267)
		G protein-coupled receptor signaling pathway (GO:0007186)
		macromolecule localization (GO:0033036)
		pigment metabolic process (GO:0042440)
		nucleobase-containing compound catabolic process (GO:0034655)
		rRNA metabolic process (GO:0016072)
		ubiquitin-dependent protein catabolic process (GO:0006511)
		macromolecule metabolic process (GO:0043170)
		small molecule biosynthetic process (GO:0044283)
		cellular protein localization (GO:0034613)
		nitrogen compound transport (GO:0071705)
		negative regulation of RNA biosynthetic process (GO:1902679)
		positive regulation of cellular amide metabolic process (GO:0034250)
		protein-containing complex assembly (GO:0065003)
		nucleotide-sugar biosynthetic process (GO:0009226)
		organic substance transport (GO:0071702)
		regulation of protein polymerization (GO:0032271)
		cellular nitrogen compound biosynthetic process (GO:0044271)
		purine ribonucleotide metabolic process (GO:0009150)
		negative regulation of transcription, DNA-templated (GO:0045892)
		organonitrogen compound metabolic process (GO:1901564)
		anion transport (GO:0006820)
		cellular component organization or biogenesis (GO:0071840)
		translation (GO:0006412)
		nucleobase-containing compound transport (GO:0015931)
		heme metabolic process (GO:0042168)
		carbohydrate derivative metabolic process (GO:1901135)
		positive regulation of immune system process (GO:0002684)
		protein folding (GO:0006457)
		maturation of SSU-rRNA (GO:0030490)
		ribonucleoprotein complex localization (GO:0071166)
		male gamete generation (GO:0048232)
		regulation of RNA biosynthetic process (GO:2001141)
		endoplasmic reticulum organization (GO:0007029)
		maturation of LSU-rRNA from tricistronic rRNA transcript (SSU-rRNA, 5.8S rRNA, LSU-rRNA) (GO:0000463)
		cellular nitrogen compound catabolic process (GO:0044270)
		regulation of cellular component size (GO:0032535)
		chemical synaptic transmission (GO:0007268)
		electron transport chain (GO:0022900)
		nucleic acid-templated transcription (GO:0097659)
		immune response-activating signal transduction (GO:0002757)
		innate immune response (GO:0045087)
		germ cell development (GO:0007281)
		cyclic-nucleotide-mediated signaling (GO:0019935)
		regulation of immune response (GO:0050776)
		proteolysis involved in cellular protein catabolic process (GO:0051603)
		regulation of cell activation (GO:0050865)
		multi-organism process (GO:0051704)
		intracellular protein transport (GO:0006886)
		cellular macromolecule catabolic process (GO:0044265)
		biological_process (GO:0008150)
		response to lipopolysaccharide (GO:0032496)
		response to topologically incorrect protein (GO:0035966)
		regulation of vesicle-mediated transport (GO:0060627)
		cell communication (GO:0007154)
		regulation of supramolecular fiber organization (GO:1902903)
		positive regulation of cell activation (GO:0050867)
		cellular aromatic compound metabolic process (GO:0006725)
		defense response to bacterium (GO:0042742)
		regulation of endocytosis (GO:0030100)
		Unclassified (UNCLASSIFIED)
		cellular respiration (GO:0045333)
		posttranscriptional regulation of gene expression (GO:0010608)
		actin polymerization or depolymerization (GO:0008154)
		regulation of mRNA metabolic process (GO:1903311)
		gene expression (GO:0010467)
		peptide transport (GO:0015833)
		purine nucleotide metabolic process (GO:0006163)
		ribonucleoprotein complex biogenesis (GO:0022613)
		cell-cell signaling (GO:0007267)
		pyrimidine nucleobase metabolic process (GO:0006206)
		protein localization (GO:0008104)
		regulation of transcription initiation from RNA polymerase II promoter (GO:0060260)
		aromatic compound catabolic process (GO:0019439)
		ATP synthesis coupled proton transport (GO:0015986)
		ATP biosynthetic process (GO:0006754)
		purine nucleoside triphosphate metabolic process (GO:0009144)
		primary metabolic process (GO:0044238)
		protein metabolic process (GO:0019538)
		biosynthetic process (GO:0009058)
		organic substance biosynthetic process (GO:1901576)
		cellular component disassembly (GO:0022411)
		cellular nitrogen compound metabolic process (GO:0034641)
		modification-dependent macromolecule catabolic process (GO:0043632)
		ribosomal large subunit biogenesis (GO:0042273)
		regulation of mRNA processing (GO:0050684)
		RNA transport (GO:0050658)
		synaptic signaling (GO:0099536)
		response to molecule of bacterial origin (GO:0002237)
		positive regulation of response to stimulus (GO:0048584)
		proteasomal protein catabolic process (GO:0010498)
		positive regulation of cellular component biogenesis (GO:0044089)
		ribosomal small subunit biogenesis (GO:0042274)
		regulation of leukocyte activation (GO:0002694)
		positive regulation of biological process (GO:0048518)
		establishment of RNA localization (GO:0051236)
		RNA splicing, via transesterification reactions with bulged adenosine as nucleophile (GO:0000377)
		humoral immune response (GO:0006959)
		peptide metabolic process (GO:0006518)
		regulation of mRNA splicing, via spliceosome (GO:0048024)
		positive regulation of lymphocyte activation (GO:0051251)
		antigen receptor-mediated signaling pathway (GO:0050851)
		ribonucleotide biosynthetic process (GO:0009260)
		protein targeting to ER (GO:0045047)
		pattern specification process (GO:0007389)
		immune response (GO:0006955)
		regulation of response to stimulus (GO:0048583)
		purine ribonucleoside triphosphate biosynthetic process (GO:0009206)
		response to external biotic stimulus (GO:0043207)
		ncRNA processing (GO:0034470)
		nucleoside triphosphate metabolic process (GO:0009141)
		retrograde vesicle-mediated transport, Golgi to endoplasmic reticulum (GO:0006890)
		nucleobase-containing compound metabolic process (GO:0006139)
		adenylate cyclase-activating G protein-coupled receptor signaling pathway (GO:0007189)
		ribosome biogenesis (GO:0042254)
		mRNA processing (GO:0006397)
		cofactor biosynthetic process (GO:0051188)
		regulation of cellular component biogenesis (GO:0044087)
		response to external stimulus (GO:0009605)
		drug metabolic process (GO:0017144)
		positive regulation of supramolecular fiber organization (GO:1902905)
		regulation of RNA polymerase II transcriptional preinitiation complex assembly (GO:0045898)
		RNA biosynthetic process (GO:0032774)
		positive regulation of actin filament polymerization (GO:0030838)
		cytosolic transport (GO:0016482)
		ncRNA metabolic process (GO:0034660)
		multi-organism reproductive process (GO:0044703)
		protein polymerization (GO:0051258)
		cellular catabolic process (GO:0044248)
		NLS-bearing protein import into nucleus (GO:0006607)
		cellular component assembly (GO:0022607)
		potassium ion transmembrane transport (GO:0071805)
		regulation of actin filament organization (GO:0110053)
		alpha-amino acid metabolic process (GO:1901605)
		cellular component organization (GO:0016043)
		macromolecule catabolic process (GO:0009057)
		ATP metabolic process (GO:0046034)
		modification-dependent protein catabolic process (GO:0019941)
		phagocytosis (GO:0006909)
		response to stimulus (GO:0050896)
		Ras protein signal transduction (GO:0007265)
		retrograde transport, endosome to Golgi (GO:0042147)
		endomembrane system organization (GO:0010256)
		response to unfolded protein (GO:0006986)
		detection of stimulus (GO:0051606)
		regulation of cellular component organization (GO:0051128)
		regulation of cytoskeleton organization (GO:0051493)
		response to organonitrogen compound (GO:0010243)
		ribonucleoprotein complex export from nucleus (GO:0071426)
		ribonucleoside triphosphate metabolic process (GO:0009199)
		oxidation-reduction process (GO:0055114)
		developmental process (GO:0032502)
		system process (GO:0003008)
		heterocycle catabolic process (GO:0046700)
		regulation of protein complex assembly (GO:0043254)
		multivesicular body sorting pathway (GO:0071985)
		cytoskeleton organization (GO:0007010)
		fatty acid catabolic process (GO:0009062)
		leukocyte activation (GO:0045321)
		transcription, DNA-templated (GO:0006351)
		regulation of macromolecule biosynthetic process (GO:0010556)
		organelle fusion (GO:0048284)
		organelle assembly (GO:0070925)
		regulation of receptor signaling pathway via STAT (GO:1904892)
		Golgi vesicle transport (GO:0048193)
		regulation of biological process (GO:0050789)
		purine-containing compound metabolic process (GO:0072521)
		COPII-coated vesicle budding (GO:0090114)
		ribose phosphate biosynthetic process (GO:0046390)
		response to toxic substance (GO:0009636)
		response to reactive oxygen species (GO:0000302)
		carboxylic acid catabolic process (GO:0046395)
		positive regulation of RNA polymerase II transcriptional preinitiation complex assembly (GO:0045899)
		actin filament organization (GO:0007015)
		positive regulation of protein complex assembly (GO:0031334)
		spermatogenesis (GO:0007283)
		cellular process involved in reproduction in multicellular organism (GO:0022412)
		generation of precursor metabolites and energy (GO:0006091)
		RNA catabolic process (GO:0006401)
		mitochondrial ATP synthesis coupled electron transport (GO:0042775)
		cellular metabolic process (GO:0044237)
		cellular macromolecule localization (GO:0070727)
		protein localization to nucleus (GO:0034504)
		protein targeting (GO:0006605)
		B cell activation (GO:0042113)
		aerobic respiration (GO:0009060)
		sensory perception (GO:0007600)
		cellular response to lipopolysaccharide (GO:0071222)
		adaptive immune response (GO:0002250)
		endoplasmic reticulum to Golgi vesicle-mediated transport (GO:0006888)
		organic substance metabolic process (GO:0071704)
		protein transport (GO:0015031)
		positive regulation of organelle organization (GO:0010638)
		regulation of transcription by RNA polymerase II (GO:0006357)
		cellular response to oxidative stress (GO:0034599)
		response to oxidative stress (GO:0006979)
		inflammatory response (GO:0006954)
		complement activation (GO:0006956)
		cellular response to molecule of bacterial origin (GO:0071219)
		small GTPase mediated signal transduction (GO:0007264)
		mRNA splicing, via spliceosome (GO:0000398)
		regulation of cellular macromolecule biosynthetic process (GO:2000112)
		cellular macromolecule metabolic process (GO:0044260)
		activation of adenylate cyclase activity (GO:0007190)
		cellular biosynthetic process (GO:0044249)
		glycolytic process (GO:0006096)
		purine nucleoside triphosphate biosynthetic process (GO:0009145)
		positive regulation of cytosolic calcium ion concentration (GO:0007204)
		establishment of localization in cell (GO:0051649)
		amide biosynthetic process (GO:0043604)
		purine nucleotide biosynthetic process (GO:0006164)
		cytoplasmic translation (GO:0002181)
		alternative mRNA splicing, via spliceosome (GO:0000380)
		cellular process (GO:0009987)
		oxidative phosphorylation (GO:0006119)
		regulation of signal transduction (GO:0009966)
		ribose phosphate metabolic process (GO:0019693)
		regulation of actin filament-based process (GO:0032970)
		B cell receptor signaling pathway (GO:0050853)
		nervous system process (GO:0050877)
		phosphate-containing compound metabolic process (GO:0006796)
		protein localization to endoplasmic reticulum (GO:0070972)
		nuclear export (GO:0051168)
		maturation of LSU-rRNA (GO:0000470)
		RNA splicing (GO:0008380)
		protein localization to organelle (GO:0033365)
		transport (GO:0006810)
		nucleoside monophosphate metabolic process (GO:0009123)
		immune response-activating cell surface receptor signaling pathway (GO:0002429)
		tetrapyrrole metabolic process (GO:0033013)
		purine ribonucleoside triphosphate metabolic process (GO:0009205)
		positive regulation of immune response (GO:0050778)
		translational initiation (GO:0006413)
		purine-containing compound biosynthetic process (GO:0072522)
		nucleotide phosphorylation (GO:0046939)
		RNA localization (GO:0006403)
		cellular modified amino acid metabolic process (GO:0006575)
		nitrogen compound metabolic process (GO:0006807)
		regulation of receptor signaling pathway via JAK-STAT (GO:0046425)
		carboxylic acid biosynthetic process (GO:0046394)
		cell fate commitment (GO:0045165)
		organic acid catabolic process (GO:0016054)
		cellular response to stress (GO:0033554)
		mRNA metabolic process (GO:0016071)
		vesicle-mediated transport (GO:0016192)
		catabolic process (GO:0009056)
		second-messenger-mediated signaling (GO:0019932)
		biological regulation (GO:0065007)
		response to biotic stimulus (GO:0009607)
		immune effector process (GO:0002252)
		supramolecular fiber organization (GO:0097435)
		organic hydroxy compound metabolic process (GO:1901615)
		energy coupled proton transport, down electrochemical gradient (GO:0015985)
		adaptive immune response based on somatic recombination of immune receptors built from immunoglobulin superfamily domains (GO:0002460)
		vacuolar transport (GO:0007034)
		signal transduction (GO:0007165)
		intra-Golgi vesicle-mediated transport (GO:0006891)
		cellular localization (GO:0051641)
		regulation of cellular process (GO:0050794)
		lymphocyte mediated immunity (GO:0002449)
		potassium ion transport (GO:0006813)
		organonitrogen compound catabolic process (GO:1901565)
		organic cyclic compound metabolic process (GO:1901360)
		anatomical structure development (GO:0048856)
		organonitrogen compound biosynthetic process (GO:1901566)
		cofactor metabolic process (GO:0051186)
		nucleoside triphosphate biosynthetic process (GO:0009142)
		cellular amino acid metabolic process (GO:0006520)
		cellular response to unfolded protein (GO:0034620)
		actin filament-based process (GO:0030029)
		spermatid development (GO:0007286)
		proton transmembrane transport (GO:1902600)
		carboxylic acid metabolic process (GO:0019752)
		organophosphate biosynthetic process (GO:0090407)
		DNA-templated transcription, initiation (GO:0006352)
		establishment of protein localization to organelle (GO:0072594)
		response to inorganic substance (GO:0010035)
		establishment of protein localization (GO:0045184)
		organelle localization (GO:0051640)
		cellular response to stimulus (GO:0051716)
		purine ribonucleotide biosynthetic process (GO:0009152)
		RNA splicing, via transesterification reactions (GO:0000375)
		small molecule catabolic process (GO:0044282)
		positive regulation of transcription initiation from RNA polymerase II promoter (GO:0060261)
		transcription by RNA polymerase II (GO:0006366)
		immunoglobulin mediated immune response (GO:0016064)
		nucleoside phosphate metabolic process (GO:0006753)
		microtubule-based process (GO:0007017)
		actin cytoskeleton organization (GO:0030036)
		rRNA processing (GO:0006364)
		proteolysis (GO:0006508)
		organelle organization (GO:0006996)
		metabolic process (GO:0008152)
		organic anion transport (GO:0015711)
		mitochondrion organization (GO:0007005)
		protein stabilization (GO:0050821)
		positive regulation of cytoskeleton organization (GO:0051495)
		chaperone-mediated protein folding (GO:0061077)
		response to endoplasmic reticulum stress (GO:0034976)
		nucleobase metabolic process (GO:0009112)
		membrane fusion (GO:0061025)
		nucleotide metabolic process (GO:0009117)
		heme biosynthetic process (GO:0006783)
		translational elongation (GO:0006414)
		protein export from nucleus (GO:0006611)
		organic acid metabolic process (GO:0006082)
		peptide biosynthetic process (GO:0043043)
		regulation of cytosolic calcium ion concentration (GO:0051480)
		regulation of protein stability (GO:0031647)
		actin filament polymerization (GO:0030041)
		cellular amide metabolic process (GO:0043603)
		regulation of cellular amide metabolic process (GO:0034248)
		localization (GO:0051179)
		regulation of transcription, DNA-templated (GO:0006355)
		phosphorus metabolic process (GO:0006793)
		ion transport (GO:0006811)
		RNA processing (GO:0006396)
		import into nucleus (GO:0051170)
		G protein-coupled receptor signaling pathway, coupled to cyclic nucleotide second messenger (GO:0007187)
		response to other organism (GO:0051707)
		protein-containing complex disassembly (GO:0032984)
		regulation of DNA-templated transcription, initiation (GO:2000142)
		trans-synaptic signaling (GO:0099537)
		sensory perception of chemical stimulus (GO:0007606)
		regulation of organelle organization (GO:0033043)
		protein-containing complex subunit organization (GO:0043933)
		vesicle fusion (GO:0006906)
		small molecule metabolic process (GO:0044281)
		negative regulation of nucleic acid-templated transcription (GO:1903507)
		ribonucleoprotein complex subunit organization (GO:0071826)
		monocarboxylic acid metabolic process (GO:0032787)
		Golgi vesicle budding (GO:0048194)
		porphyrin-containing compound metabolic process (GO:0006778)
		cellular component biogenesis (GO:0044085)
		cellular response to biotic stimulus (GO:0071216)
		positive regulation of leukocyte activation (GO:0002696)
		regulation of translation (GO:0006417)
		multicellular organismal process (GO:0032501)
		regulation of actin cytoskeleton organization (GO:0032956)
		cell surface receptor signaling pathway (GO:0007166)
		immune response-regulating cell surface receptor signaling pathway (GO:0002768)
		nucleotide biosynthetic process (GO:0009165)
		endosome transport via multivesicular body sorting pathway (GO:0032509)
		organic substance catabolic process (GO:1901575)
		regulation of microtubule-based process (GO:0032886)
		cellular protein-containing complex assembly (GO:0034622)
		sulfur compound metabolic process (GO:0006790)
		ribonucleoprotein complex assembly (GO:0022618)
		protein catabolic process (GO:0030163)
		positive regulation of DNA-templated transcription, initiation (GO:2000144)
		positive regulation of cellular component organization (GO:0051130)
		RNA export from nucleus (GO:0006405)
		tricarboxylic acid cycle (GO:0006099)
		regulation of alternative mRNA splicing, via spliceosome (GO:0000381)
		energy derivation by oxidation of organic compounds (GO:0015980)
		ATP synthesis coupled electron transport (GO:0042773)
		respiratory electron transport chain (GO:0022904)
		proteasome-mediated ubiquitin-dependent protein catabolic process (GO:0043161)
		ribosomal large subunit assembly (GO:0000027)
		ribonucleoside triphosphate biosynthetic process (GO:0009201)
		regulation of membrane potential (GO:0042391)
		nucleic acid transport (GO:0050657)
		metal ion transport (GO:0030001)
		nucleocytoplasmic transport (GO:0006913)
		protein import (GO:0017038)
		amide transport (GO:0042886)
		establishment of protein localization to endoplasmic reticulum (GO:0072599)
		regulation of lymphocyte activation (GO:0051249)
		regulation of nucleic acid-templated transcription (GO:1903506)
		cellular protein complex disassembly (GO:0043624)
		cell activation (GO:0001775)
		positive regulation of translation (GO:0045727)
		positive regulation of protein polymerization (GO:0032273)
		pre-replicative complex assembly involved in nuclear cell cycle DNA replication (GO:0006267)
		defense response (GO:0006952)
		immune response-regulating signaling pathway (GO:0002764)
		intracellular transport (GO:0046907)
		transcription initiation from RNA polymerase II promoter (GO:0006367)
		cellular response to topologically incorrect protein (GO:0035967)
		cellular protein catabolic process (GO:0044257)
		organic cyclic compound catabolic process (GO:1901361)
		response to bacterium (GO:0009617)
		regulation of RNA splicing (GO:0043484)
		activation of immune response (GO:0002253)
		regulation of actin filament length (GO:0030832)
		mRNA cis splicing, via spliceosome (GO:0045292)
		establishment of localization (GO:0051234)
		heterocycle metabolic process (GO:0046483)
		nucleobase-containing small molecule metabolic process (GO:0055086)
		regulation of immune system process (GO:0002682)
		reactive oxygen species metabolic process (GO:0072593)
		lymphocyte activation (GO:0046649)
		defense response to other organism (GO:0098542)
		nucleoside phosphate biosynthetic process (GO:1901293)
		organophosphate metabolic process (GO:0019637)
		endosomal transport (GO:0016197)
		Arp2/3 complex-mediated actin nucleation (GO:0034314)
		antimicrobial humoral immune response mediated by antimicrobial peptide (GO:0061844)
		signaling (GO:0023052)
		ERAD pathway (GO:0036503)
		ribonucleotide metabolic process (GO:0009259)
		oxoacid metabolic process (GO:0043436)
ND BP	69	ribosome localization (GO:0033750)
		negative regulation of biosynthetic process (GO:0009890)
		mitotic cell cycle process (GO:1903047)
		multicellular organism development (GO:0007275)
		meiotic cell cycle (GO:0051321)
		mRNA transport (GO:0051028)
		regulation of cAMP-mediated signaling (GO:0043949)
		positive regulation of transcription, DNA-templated (GO:0045893)
		vesicle targeting, trans-Golgi to periciliary membrane compartment (GO:0097712)
		protein localization to cilium (GO:0061512)
		negative regulation of cellular macromolecule biosynthetic process (GO:2000113)
		protein transport along microtubule (GO:0098840)
		rRNA-containing ribonucleoprotein complex export from nucleus (GO:0071428)
		negative regulation of cellular biosynthetic process (GO:0031327)
		positive regulation of RNA biosynthetic process (GO:1902680)
		cytokine-mediated signaling pathway (GO:0019221)
		negative regulation of macromolecule biosynthetic process (GO:0010558)
		negative regulation of nitrogen compound metabolic process (GO:0051172)
		ciliary transition zone assembly (GO:1905349)
		reproduction (GO:0000003)
		reproductive process (GO:0022414)
		coenzyme metabolic process (GO:0006732)
		coenzyme biosynthetic process (GO:0009108)
		regulation of RNA metabolic process (GO:0051252)
		intraciliary transport (GO:0042073)
		response to lipid (GO:0033993)
		meiotic cell cycle process (GO:1903046)
		negative regulation of nucleobase-containing compound metabolic process (GO:0045934)
		negative regulation of transcription by RNA polymerase II (GO:0000122)
		axoneme assembly (GO:0035082)
		negative regulation of translation (GO:0017148)
		establishment of protein localization to vacuole (GO:0072666)
		negative regulation of RNA metabolic process (GO:0051253)
		mitotic nuclear division (GO:0140014)
		microtubule cytoskeleton organization involved in mitosis (GO:1902850)
		meiotic nuclear division (GO:0140013)
		positive regulation of cellular process (GO:0048522)
		cortical cytoskeleton organization (GO:0030865)
		positive regulation of nucleic acid-templated transcription (GO:1903508)
		establishment or maintenance of cell polarity (GO:0007163)
		mRNA export from nucleus (GO:0006406)
		ribosomal subunit export from nucleus (GO:0000054)
		maturation of SSU-rRNA from tricistronic rRNA transcript (SSU-rRNA, 5.8S rRNA, LSU-rRNA) (GO:0000462)
		cellular response to lipid (GO:0071396)
		cilium assembly (GO:0060271)
		cAMP-mediated signaling (GO:0019933)
		cristae formation (GO:0042407)
		mitotic cell cycle (GO:0000278)
		receptor signaling pathway via JAK-STAT (GO:0007259)
		cell chemotaxis (GO:0060326)
		receptor signaling pathway via STAT (GO:0097696)
		inositol phosphate-mediated signaling (GO:0048016)
		Golgi organization (GO:0007030)
		cilium organization (GO:0044782)
		regulation of cellular biosynthetic process (GO:0031326)
		protein modification by small protein conjugation or removal (GO:0070647)
		late endosome to vacuole transport (GO:0045324)
		intraciliary transport involved in cilium assembly (GO:0035735)
		positive regulation of transcription by RNA polymerase II (GO:0045944)
		regulation of nucleobase-containing compound metabolic process (GO:0019219)
		negative regulation of cellular metabolic process (GO:0031324)
		positive regulation of RNA metabolic process (GO:0051254)
		regulation of biosynthetic process (GO:0009889)
		mRNA-containing ribonucleoprotein complex export from nucleus (GO:0071427)
		microtubule-based protein transport (GO:0099118)
		positive regulation of lipase activity (GO:0060193)
		embryo development (GO:0009790)
		microtubule bundle formation (GO:0001578)
		positive regulation of nucleobase-containing compound metabolic process (GO:0045935)
HFD BP	30	regulation of cell communication (GO:0010646)
		regulation of RNA stability (GO:0043487)
		regulation of translational initiation (GO:0006446)
		regionalization (GO:0003002)
		RNA metabolic process (GO:0016070)
		animal organ development (GO:0048513)
		RNA 3'-end processing (GO:0031123)
		regulation of signaling (GO:0023051)
		Golgi to plasma membrane transport (GO:0006893)
		mitochondrial electron transport, ubiquinol to cytochrome c (GO:0006122)
		mRNA 3'-end processing (GO:0031124)
		regulation of mRNA catabolic process (GO:0061013)
		post-Golgi vesicle-mediated transport (GO:0006892)
		glutathione metabolic process (GO:0006749)
		pyrimidine-containing compound metabolic process (GO:0072527)
		regulation of mRNA stability (GO:0043488)
		positive regulation of cell population proliferation (GO:0008284)
		ubiquitin-dependent ERAD pathway (GO:0030433)
		response to nitrogen compound (GO:1901698)
		IMP metabolic process (GO:0046040)
		nucleobase-containing small molecule biosynthetic process (GO:0034404)
		RNA phosphodiester bond hydrolysis, endonucleolytic (GO:0090502)
		spliceosomal snRNP assembly (GO:0000387)
		pyrimidine nucleotide metabolic process (GO:0006220)
		microtubule cytoskeleton organization (GO:0000226)
		cellular response to organonitrogen compound (GO:0071417)
		double-strand break repair via break-induced replication (GO:0000727)
		import into cell (GO:0098657)
		nucleic acid metabolic process (GO:0090304)
		mRNA catabolic process (GO:0006402)

Names	total	elements		
HFD MF ND MF	128	RNA binding (GO:0003723)		
		secondary active transmembrane transporter activity (GO:0015291)		
		voltage-gated cation channel activity (GO:0022843)		
		receptor ligand activity (GO:0048018)		
		transition metal ion binding (GO:0046914)		
		polyubiquitin modification-dependent protein binding (GO:0031593)		
		ATPase activity (GO:0016887)		
		translation initiation factor activity (GO:0003743)		
		signaling receptor binding (GO:0005102)		
		translation initiation factor binding (GO:0031369)		
		carbon-oxygen lyase activity (GO:0016835)		
		heat shock protein binding (GO:0031072)		
		ATP binding (GO:0005524)		
		lyase activity (GO:0016829)		
		ion channel activity (GO:0005216)		
		sequence-specific double-stranded DNA binding (GO:1990837)		
		single-stranded RNA binding (GO:0003727)		
		molecular_function (GO:0003674)		
		regulatory region nucleic acid binding (GO:0001067)		
		ion gated channel activity (GO:0022839)		
		actin binding (GO:0003779)		
		small molecule binding (GO:0036094)		
		catalytic activity, acting on a protein (GO:0140096)		
		metal ion binding (GO:0046872)		
		carbohydrate derivative binding (GO:0097367)		
		ribosome binding (GO:0043022)		
		drug binding (GO:0008144)		
		ATPase activity, coupled (GO:0042623)		
		nucleoside-triphosphatase regulator activity (GO:0060589)		
		signal sequence binding (GO:0005048)		
		ligase activity, forming carbon-sulfur bonds (GO:0016877)		
		DNA-binding transcription factor activity, RNA polymerase II-specific (GO:0000981)		
		catalytic activity, acting on RNA (GO:0140098)		
		G protein-coupled receptor activity (GO:0004930)		
		hydro-lyase activity (GO:0016836)		
		hydrolase activity, acting on acid anhydrides, in phosphorus-containing anhydrides (GO:0016818)		
		DNA binding (GO:0003677)		
		protein phosphatase binding (GO:0019903)		
		peptidase activity, acting on L-amino acid peptides (GO:0070011)		
		pyrophosphatase activity (GO:0016462)		
		peptide receptor activity (GO:0001653)		
		transmembrane transporter activity (GO:0022857)		
		isomerase activity (GO:0016853)		
		RNA polymerase II proximal promoter sequence-specific DNA binding (GO:0000978)		
		enzyme regulator activity (GO:0030234)		
		hydrolase activity, acting on acid anhydrides (GO:0016817)		
		voltage-gated potassium channel activity (GO:0005249)		
		G protein-coupled peptide receptor activity (GO:0008528)		
		ligase activity (GO:0016874)		
		ion binding (GO:0043167)		
		GTPase regulator activity (GO:0030695)		
		cytokine activity (GO:0005125)		
		metal ion transmembrane transporter activity (GO:0046873)		
		potassium ion transmembrane transporter activity (GO:0015079)		
		inorganic molecular entity transmembrane transporter activity (GO:0015318)		
		phosphatase binding (GO:0019902)		
		nucleoside-triphosphatase activity (GO:0017111)		
		ribonucleotide binding (GO:0032553)		
		organic anion transmembrane transporter activity (GO:0008514)		
		ubiquitin binding (GO:0043130)		
		hydrolase activity (GO:0016787)		
		GTPase activity (GO:0003924)		
		heterocyclic compound binding (GO:1901363)		
		double-stranded DNA binding (GO:0003690)		
		signaling receptor activator activity (GO:0030546)		
		purine nucleotide binding (GO:0017076)		
		transcription regulator activity (GO:0140110)		
		DNA-binding transcription factor activity (GO:0003700)		
		potassium channel activity (GO:0005267)		
		mRNA binding (GO:0003729)		
		methylated histone binding (GO:0035064)		
		oxidoreductase activity (GO:0016491)		
		ion transmembrane transporter activity (GO:0015075)		
		transmembrane signaling receptor activity (GO:0004888)		
		Unclassified (UNCLASSIFIED)		
		structural constituent of nuclear pore (GO:0017056)		
		catalytic activity (GO:0003824)		
		ribonucleoprotein complex binding (GO:0043021)		
		single-stranded DNA binding (GO:0003697)		
		transcription regulatory region sequence-specific DNA binding (GO:0000976)		
		neurotransmitter receptor activity (GO:0030594)		
		rRNA binding (GO:0019843)		
		gated channel activity (GO:0022836)		
		cytoskeletal protein binding (GO:0008092)		
		cation channel activity (GO:0005261)		
		channel activity (GO:0015267)		
		signaling receptor activity (GO:0038023)		
		cofactor binding (GO:0048037)		
		structural constituent of ribosome (GO:0003735)		
		molecular transducer activity (GO:0060089)		
		neurotransmitter binding (GO:0042165)		
		passive transmembrane transporter activity (GO:0022803)		
		electron transfer activity (GO:0009055)		
		protein-containing complex binding (GO:0044877)		
		sequence-specific DNA binding (GO:0043565)		
		receptor regulator activity (GO:0030545)		
		RNA polymerase II regulatory region DNA binding (GO:0001012)		
		unfolded protein binding (GO:0051082)		
		RNA polymerase II regulatory region sequence-specific DNA binding (GO:0000977)		
		cytokine binding (GO:0019955)		
		cation transmembrane transporter activity (GO:0008324)		
		GTP binding (GO:0005525)		
		nucleoside phosphate binding (GO:1901265)		
		oxidoreductase activity, acting on the CH-OH group of donors, NAD or NADP as acceptor (GO:0016616)		
		inorganic cation transmembrane transporter activity (GO:0022890)		
		transporter activity (GO:0005215)		
		hormone activity (GO:0005179)		
		actin filament binding (GO:0051015)		
		enzyme binding (GO:0019899)		
		anion binding (GO:0043168)		
		ubiquitin-like modifier activating enzyme activity (GO:0008641)		
		peptidase activity (GO:0008233)		
		purine ribonucleotide binding (GO:0032555)		
		nucleotide binding (GO:0000166)		
		structural molecule activity (GO:0005198)		
		chaperone binding (GO:0051087)		
		binding (GO:0005488)		
		transcription regulatory region DNA binding (GO:0044212)		
		DNA-binding transcription activator activity, RNA polymerase II-specific (GO:0001228)		
		purine ribonucleoside triphosphate binding (GO:0035639)		
		nucleic acid binding (GO:0003676)		
		protein binding (GO:0005515)		
		mRNA 3'-UTR binding (GO:0003730)		
		organic cyclic compound binding (GO:0097159)		
		DNA-binding transcription activator activity (GO:0001216)		
		translation regulator activity (GO:0045182)		
		cytokine receptor binding (GO:0005126)		
ND MF	13	RNA polymerase activity (GO:0097747)		
		voltage-gated ion channel activity (GO:0005244)		
		sterol binding (GO:0032934)		
		voltage-gated channel activity (GO:0022832)		
		carbohydrate binding (GO:0030246)		
		monooxygenase activity (GO:0004497)		
		NADH dehydrogenase activity (GO:0003954)		
		steroid hydroxylase activity (GO:0008395)		
		G protein-coupled receptor binding (GO:0001664)		
		carbohydrate kinase activity (GO:0019200)		
		5'-3' RNA polymerase activity (GO:0034062)		
		transferase activity (GO:0016740)		
		DNA-directed 5'-3' RNA polymerase activity (GO:0003899)		
HFD MF	4	proton transmembrane transporter activity (GO:0015078)		
		anion transmembrane transporter activity (GO:0008509)		
		DNA-dependent ATPase activity (GO:0008094)		
		snRNA binding (GO:0017069)		

Names	total	elements		
HFD BP ND BP	234	complement activation (GO:0006956)		
		immune system process (GO:0002376)		
		mRNA splicing, via spliceosome (GO:0000398)		
		regulation of anatomical structure size (GO:0090066)		
		regulation of cellular macromolecule biosynthetic process (GO:2000112)		
		cellular macromolecule metabolic process (GO:0044260)		
		organic cyclic compound biosynthetic process (GO:1901362)		
		locomotion (GO:0040011)		
		angiogenesis (GO:0001525)		
		leukocyte mediated immunity (GO:0002443)		
		regulation of actin polymerization or depolymerization (GO:0008064)		
		regulation of actin filament polymerization (GO:0030833)		
		regulation of peptidase activity (GO:0052547)		
		cell motility (GO:0048870)		
		cellular protein metabolic process (GO:0044267)		
		glycolytic process (GO:0006096)		
		G protein-coupled receptor signaling pathway (GO:0007186)		
		anatomical structure morphogenesis (GO:0009653)		
		amide biosynthetic process (GO:0043604)		
		extracellular matrix organization (GO:0030198)		
		ubiquitin-dependent protein catabolic process (GO:0006511)		
		growth (GO:0040007)		
		cytoplasmic translation (GO:0002181)		
		neuron projection development (GO:0031175)		
		cellular process (GO:0009987)		
		aromatic compound biosynthetic process (GO:0019438)		
		regulation of actin filament-based process (GO:0032970)		
		negative regulation of protein metabolic process (GO:0051248)		
		macromolecule metabolic process (GO:0043170)		
		small molecule biosynthetic process (GO:0044283)		
		B cell receptor signaling pathway (GO:0050853)		
		extracellular structure organization (GO:0043062)		
		cell migration (GO:0016477)		
		nervous system process (GO:0050877)		
		negative regulation of RNA biosynthetic process (GO:1902679)		
		protein-containing complex assembly (GO:0065003)		
		axo-dendritic transport (GO:0008088)		
		tube morphogenesis (GO:0035239)		
		cellular lipid metabolic process (GO:0044255)		
		cell projection morphogenesis (GO:0048858)		
		RNA splicing (GO:0008380)		
		regulation of protein polymerization (GO:0032271)		
		regulation of biological quality (GO:0065008)		
		multicellular organism development (GO:0007275)		
		negative regulation of transcription, DNA-templated (GO:0045892)		
		muscle structure development (GO:0061061)		
		organonitrogen compound metabolic process (GO:1901564)		
		immune response-activating cell surface receptor signaling pathway (GO:0002429)		
		positive regulation of immune response (GO:0050778)		
		translation (GO:0006412)		
		cellular component organization or biogenesis (GO:0071840)		
		translational initiation (GO:0006413)		
		tube development (GO:0035295)		
		protein folding (GO:0006457)		
		nucleotide phosphorylation (GO:0046939)		
		nitrogen compound metabolic process (GO:0006807)		
		IMP metabolic process (GO:0046040)		
		regulation of RNA biosynthetic process (GO:2001141)		
		regulation of cellular component size (GO:0032535)		
		neurogenesis (GO:0022008)		
		nucleic acid-templated transcription (GO:0097659)		
		catabolic process (GO:0009056)		
		immune response-activating signal transduction (GO:0002757)		
		negative regulation of actin filament polymerization (GO:0030837)		
		axon development (GO:0061564)		
		supramolecular fiber organization (GO:0097435)		
		proteolysis involved in cellular protein catabolic process (GO:0051603)		
		regulation of immune response (GO:0050776)		
		multi-organism process (GO:0051704)		
		adaptive immune response based on somatic recombination of immune receptors built from immunoglobulin superfamily d		
		cellular macromolecule catabolic process (GO:0044265)		
		biological_process (GO:0008150)		
		anatomical structure formation involved in morphogenesis (GO:0048646)		
		developmental growth (GO:0048589)		
		cell development (GO:0048468)		
		signal transduction (GO:0007165)		
		cell communication (GO:0007154)		
		negative regulation of endopeptidase activity (GO:0010951)		
		regulation of supramolecular fiber organization (GO:1902903)		
		lymphocyte mediated immunity (GO:0002449)		
		cell differentiation (GO:0030154)		
		Unclassified (UNCLASSIFIED)		
		organonitrogen compound catabolic process (GO:1901565)		
		posttranscriptional regulation of gene expression (GO:0010608)		
		actin polymerization or depolymerization (GO:0008154)		
		anatomical structure development (GO:0048856)		
		organonitrogen compound biosynthetic process (GO:1901566)		
		proteasome assembly (GO:0043248)		
		purine nucleotide metabolic process (GO:0006163)		
		ribonucleoprotein complex biogenesis (GO:0022613)		
		regulation of cellular protein metabolic process (GO:0032268)		
		positive chemotaxis (GO:0050918)		
		sprouting angiogenesis (GO:0002040)		
		generation of neurons (GO:0048699)		
		actin filament-based process (GO:0030029)		
		system development (GO:0048731)		
		primary metabolic process (GO:0044238)		
		protein metabolic process (GO:0019538)		
		actin filament depolymerization (GO:0030042)		
		ribosomal large subunit biogenesis (GO:0042273)		
		modification-dependent macromolecule catabolic process (GO:0043632)		
		response to inorganic substance (GO:0010035)		
		cellular response to stimulus (GO:0051716)		
		movement of cell or subcellular component (GO:0006928)		
		RNA splicing, via transesterification reactions (GO:0000375)		
		transcription by RNA polymerase II (GO:0006366)		
		immunoglobulin mediated immune response (GO:0016064)		
		proteasomal protein catabolic process (GO:0010498)		
		ribosomal small subunit biogenesis (GO:0042274)		
		positive regulation of cellular component biogenesis (GO:0044089)		
		microtubule-based process (GO:0007017)		
		RNA splicing, via transesterification reactions with bulged adenosine as nucleophile (GO:0000377)		
		actin cytoskeleton organization (GO:0030036)		
		vasculature development (GO:0001944)		
		humoral immune response (GO:0006959)		
		proteolysis (GO:0006508)		
		organelle organization (GO:0006996)		
		peptide metabolic process (GO:0006518)		
		metabolic process (GO:0008152)		
		regulation of mRNA splicing, via spliceosome (GO:0048024)		
		protein stabilization (GO:0050821)		
		positive regulation of cytoskeleton organization (GO:0051495)		
		chaperone-mediated protein folding (GO:0061077)		
		nucleobase metabolic process (GO:0009112)		
		cell morphogenesis involved in neuron differentiation (GO:0048667)		
		animal organ morphogenesis (GO:0009887)		
		antigen receptor-mediated signaling pathway (GO:0050851)		
		cell part morphogenesis (GO:0032990)		
		regulation of protein metabolic process (GO:0051246)		
		protein depolymerization (GO:0051261)		
		immune response (GO:0006955)		
		translational elongation (GO:0006414)		
		neuron development (GO:0048666)		
		peptide biosynthetic process (GO:0043043)		
		regulation of protein stability (GO:0031647)		
		regulation of transcription, DNA-templated (GO:0006355)		
		cellular amide metabolic process (GO:0043603)		
		actin filament polymerization (GO:0030041)		
		ion transport (GO:0006811)		
		localization of cell (GO:0051674)		
		cell morphogenesis involved in differentiation (GO:0000904)		
		protein-containing complex disassembly (GO:0032984)		
		dendrite development (GO:0016358)		
		ribosome biogenesis (GO:0042254)		
		negative regulation of proteolysis (GO:0045861)		
		regulation of cellular component biogenesis (GO:0044087)		
		sensory perception of chemical stimulus (GO:0007606)		
		regulation of organelle organization (GO:0033043)		
		protein-containing complex subunit organization (GO:0043933)		
		positive regulation of supramolecular fiber organization (GO:1902905)		
		negative regulation of nucleic acid-templated transcription (GO:1903507)		
		small molecule metabolic process (GO:0044281)		
		ribonucleoprotein complex subunit organization (GO:0071826)		
		RNA biosynthetic process (GO:0032774)		
		positive regulation of actin filament polymerization (GO:0030838)		
		tissue development (GO:0009888)		
		cellular component biogenesis (GO:0044085)		
		protein polymerization (GO:0051258)		
		cellular catabolic process (GO:0044248)		
		regulation of translation (GO:0006417)		
		circulatory system development (GO:0072359)		
		cellular component assembly (GO:0022607)		
		blood vessel development (GO:0001568)		
		regulation of actin cytoskeleton organization (GO:0032956)		
		negative regulation of hydrolase activity (GO:0051346)		
		immune response-regulating cell surface receptor signaling pathway (GO:0002768)		
		regulation of actin filament organization (GO:0110053)		
		cellular component organization (GO:0016043)		
		macromolecule catabolic process (GO:0009057)		
		modification-dependent protein catabolic process (GO:0019941)		
		organic substance catabolic process (GO:1901575)		
		nucleobase-containing compound biosynthetic process (GO:0034654)		
		response to stimulus (GO:0050896)		
		cellular protein-containing complex assembly (GO:0034622)		
		neuron differentiation (GO:0030182)		
		ribonucleoprotein complex assembly (GO:0022618)		
		protein catabolic process (GO:0030163)		
		regulation of cellular component organization (GO:0051128)		
		regulation of cytoskeleton organization (GO:0051493)		
		positive regulation of cellular component organization (GO:0051130)		
		response to hypoxia (GO:0001666)		
		cell morphogenesis (GO:0000902)		
		oxidation-reduction process (GO:0055114)		
		developmental process (GO:0032502)		
		regulation of protein complex assembly (GO:0043254)		
		cytoskeleton organization (GO:0007010)		
		heterocycle biosynthetic process (GO:0018130)		
		transcription, DNA-templated (GO:0006351)		
		proteasome-mediated ubiquitin-dependent protein catabolic process (GO:0043161)		
		organelle assembly (GO:0070925)		
		ribosomal large subunit assembly (GO:0000027)		
		purine-containing compound metabolic process (GO:0072521)		
		regulation of lymphocyte activation (GO:0051249)		
		negative regulation of cellular protein metabolic process (GO:0032269)		
		regulation of nucleic acid-templated transcription (GO:1903506)		
		negative regulation of peptidase activity (GO:0010466)		
		response to toxic substance (GO:0009636)		
		cellular protein complex disassembly (GO:0043624)		
		positive regulation of protein polymerization (GO:0032273)		
		cellular component morphogenesis (GO:0032989)		
		response to reactive oxygen species (GO:0000302)		
		negative regulation of cellular component organization (GO:0051129)		
		actin filament organization (GO:0007015)		
		collagen fibril organization (GO:0030199)		
		regulation of synapse organization (GO:0050807)		
		immune response-regulating signaling pathway (GO:0002764)		
		positive regulation of protein complex assembly (GO:0031334)		
		cellular protein catabolic process (GO:0044257)		
		regulation of endopeptidase activity (GO:0052548)		
		generation of precursor metabolites and energy (GO:0006091)		
		response to bacterium (GO:0009617)		
		cellular metabolic process (GO:0044237)		
		regulation of RNA splicing (GO:0043484)		
		activation of immune response (GO:0002253)		
		regulation of actin filament length (GO:0030832)		
		blood vessel morphogenesis (GO:0048514)		
		nucleobase-containing small molecule metabolic process (GO:0055086)		
		B cell activation (GO:0042113)		
		sensory perception (GO:0007600)		
		adaptive immune response (GO:0002250)		
		lymphocyte activation (GO:0046649)		
		reactive oxygen species metabolic process (GO:0072593)		
		organic substance metabolic process (GO:0071704)		
		axonogenesis (GO:0007409)		
		Arp2/3 complex-mediated actin nucleation (GO:0034314)		
		cardiovascular system development (GO:0072358)		
		neuron projection morphogenesis (GO:0048812)		
		positive regulation of organelle organization (GO:0010638)		
		signaling (GO:0023052)		
		regulation of transcription by RNA polymerase II (GO:0006357)		
		cellular response to oxidative stress (GO:0034599)		
		plasma membrane bounded cell projection morphogenesis (GO:0120039)		
		response to oxidative stress (GO:0006979)		
ND BP	50	positive regulation of locomotion (GO:0040017)		
		negative regulation of catalytic activity (GO:0043086)		
		regulation of anatomical structure morphogenesis (GO:0022603)		
		positive regulation of cellular component movement (GO:0051272)		
		organelle transport along microtubule (GO:0072384)		
		biological regulation (GO:0065007)		
		positive regulation of transcription, DNA-templated (GO:0045893)		
		positive regulation of nucleic acid-templated transcription (GO:1903508)		
		nucleus localization (GO:0051647)		
		RNA metabolic process (GO:0016070)		
		myeloid leukocyte migration (GO:0097529)		
		nuclear migration (GO:0007097)		
		leukocyte chemotaxis (GO:0030595)		
		carboxylic acid metabolic process (GO:0019752)		
		cellular component disassembly (GO:0022411)		
		muscle contraction (GO:0006936)		
		regulation of microtubule polymerization or depolymerization (GO:0031110)		
		nucleoside phosphate metabolic process (GO:0006753)		
		enzyme linked receptor protein signaling pathway (GO:0007167)		
		positive regulation of RNA biosynthetic process (GO:1902680)		
		response to endoplasmic reticulum stress (GO:0034976)		
		neuron projection guidance (GO:0097485)		
		nucleotide metabolic process (GO:0009117)		
		microtubule cytoskeleton organization (GO:0000226)		
		organic acid metabolic process (GO:0006082)		
		leukocyte migration (GO:0050900)		
		cellular developmental process (GO:0048869)		
		axon guidance (GO:0007411)		
		chemotaxis (GO:0006935)		
		positive regulation of macromolecule biosynthetic process (GO:0010557)		
		regulation of cellular biosynthetic process (GO:0031326)		
		cell projection organization (GO:0030030)		
		nucleic acid metabolic process (GO:0090304)		
		positive regulation of cell motility (GO:2000147)		
		regulation of macromolecule biosynthetic process (GO:0010556)		
		regulation of biological process (GO:0050789)		
		regulation of gene expression (GO:0010468)		
		positive regulation of transcription by RNA polymerase II (GO:0045944)		
		regulation of nucleobase-containing compound metabolic process (GO:0019219)		
		nervous system development (GO:0007399)		
		regulation of RNA metabolic process (GO:0051252)		
		positive regulation of RNA metabolic process (GO:0051254)		
		regulation of biosynthetic process (GO:0009889)		
		negative regulation of nucleobase-containing compound metabolic process (GO:0045934)		
		regulation of cell motility (GO:2000145)		
		taxis (GO:0042330)		
		positive regulation of cell migration (GO:0030335)		
		ribonucleotide metabolic process (GO:0009259)		
		oxoacid metabolic process (GO:0043436)		
		positive regulation of nucleobase-containing compound metabolic process (GO:0045935)		
HFD BP	40	protein import into nucleus (GO:0006606)		
		transmembrane transport (GO:0055085)		
		alternative mRNA splicing, via spliceosome (GO:0000380)		
		intracellular signal transduction (GO:0035556)		
		maturation of SSU-rRNA (GO:0030490)		
		cation transport (GO:0006812)		
		mRNA metabolic process (GO:0016071)		
		response to biotic stimulus (GO:0009607)		
		innate immune response (GO:0045087)		
		immune effector process (GO:0002252)		
		intra-Golgi vesicle-mediated transport (GO:0006891)		
		defense response to bacterium (GO:0042742)		
		ion transmembrane transport (GO:0034220)		
		regulation of mRNA metabolic process (GO:1903311)		
		regulation of transcription initiation from RNA polymerase II promoter (GO:0060260)		
		peptidyl-lysine modification (GO:0018205)		
		cellular nitrogen compound metabolic process (GO:0034641)		
		regulation of mRNA processing (GO:0050684)		
		regulation of proteolysis (GO:0030162)		
		positive regulation of transcription initiation from RNA polymerase II promoter (GO:0060261)		
		bone morphogenesis (GO:0060349)		
		positive regulation of lymphocyte activation (GO:0051251)		
		regulation of cellular amide metabolic process (GO:0034248)		
		response to external biotic stimulus (GO:0043207)		
		RNA processing (GO:0006396)		
		import into nucleus (GO:0051170)		
		response to other organism (GO:0051707)		
		regulation of DNA-templated transcription, initiation (GO:2000142)		
		mRNA processing (GO:0006397)		
		regulation of transmembrane receptor protein serine/threonine kinase signaling pathway (GO:0090092)		
		regulation of RNA polymerase II transcriptional preinitiation complex assembly (GO:0045898)		
		negative regulation of organelle organization (GO:0010639)		
		positive regulation of DNA-templated transcription, initiation (GO:2000144)		
		regulation of alternative mRNA splicing, via spliceosome (GO:0000381)		
		defense response (GO:0006952)		
		positive regulation of RNA polymerase II transcriptional preinitiation complex assembly (GO:0045899)		
		transcription initiation from RNA polymerase II promoter (GO:0006367)		
		regulation of immune system process (GO:0002682)		
		defense response to other organism (GO:0098542)		
		RNA polymerase II preinitiation complex assembly (GO:0051123)		

Names	total	elements		
HFD BP ND BP	85	RNA binding (GO:0003723)		
		ribonucleotide binding (GO:0032553)		
		hydrolase activity (GO:0016787)		
		extracellular matrix structural constituent (GO:0005201)		
		heterocyclic compound binding (GO:1901363)		
		double-stranded DNA binding (GO:0003690)		
		cell adhesion molecule binding (GO:0050839)		
		purine nucleotide binding (GO:0017076)		
		translation initiation factor activity (GO:0003743)		
		transcription regulator activity (GO:0140110)		
		DNA-binding transcription factor activity (GO:0003700)		
		mRNA binding (GO:0003729)		
		extracellular matrix binding (GO:0050840)		
		oxidoreductase activity (GO:0016491)		
		ion transmembrane transporter activity (GO:0015075)		
		transmembrane signaling receptor activity (GO:0004888)		
		integrin binding (GO:0005178)		
		Unclassified (UNCLASSIFIED)		
		catalytic activity (GO:0003824)		
		ribonucleoprotein complex binding (GO:0043021)		
		oxidoreductase activity, acting on peroxide as acceptor (GO:0016684)		
		molecular_function (GO:0003674)		
		heparin binding (GO:0008201)		
		serine hydrolase activity (GO:0017171)		
		regulatory region nucleic acid binding (GO:0001067)		
		rRNA binding (GO:0019843)		
		endopeptidase activity (GO:0004175)		
		cytoskeletal protein binding (GO:0008092)		
		cation channel activity (GO:0005261)		
		actin binding (GO:0003779)		
		small molecule binding (GO:0036094)		
		signaling receptor activity (GO:0038023)		
		catalytic activity, acting on a protein (GO:0140096)		
		metal ion binding (GO:0046872)		
		carbohydrate derivative binding (GO:0097367)		
		ribosome binding (GO:0043022)		
		drug binding (GO:0008144)		
		molecular transducer activity (GO:0060089)		
		structural constituent of ribosome (GO:0003735)		
		protein-containing complex binding (GO:0044877)		
		DNA-binding transcription factor activity, RNA polymerase II-specific (GO:0000981)		
		G protein-coupled receptor activity (GO:0004930)		
		sequence-specific DNA binding (GO:0043565)		
		RNA polymerase II regulatory region DNA binding (GO:0001012)		
		enzyme inhibitor activity (GO:0004857)		
		RNA polymerase II regulatory region sequence-specific DNA binding (GO:0000977)		
		unfolded protein binding (GO:0051082)		
		cation transmembrane transporter activity (GO:0008324)		
		protease binding (GO:0002020)		
		metalloendopeptidase activity (GO:0004222)		
		oxidoreductase activity, acting on the CH-OH group of donors, NAD or NADP as acceptor (GO:0016616)		
		nucleoside phosphate binding (GO:1901265)		
		inorganic cation transmembrane transporter activity (GO:0022890)		
		peptidase inhibitor activity (GO:0030414)		
		transporter activity (GO:0005215)		
		endopeptidase inhibitor activity (GO:0004866)		
		actin filament binding (GO:0051015)		
		anion binding (GO:0043168)		
		DNA binding (GO:0003677)		
		peptidase regulator activity (GO:0061134)		
		serine-type endopeptidase activity (GO:0004252)		
		peptidase activity (GO:0008233)		
		peptidase activity, acting on L-amino acid peptides (GO:0070011)		
		glycosaminoglycan binding (GO:0005539)		
		purine ribonucleotide binding (GO:0032555)		
		transmembrane transporter activity (GO:0022857)		
		isomerase activity (GO:0016853)		
		enzyme regulator activity (GO:0030234)		
		nucleotide binding (GO:0000166)		
		structural molecule activity (GO:0005198)		
		hydrolase activity, acting on acid phosphorus-nitrogen bonds (GO:0016825)		
		binding (GO:0005488)		
		ligase activity (GO:0016874)		
		transcription regulatory region DNA binding (GO:0044212)		
		purine ribonucleoside triphosphate binding (GO:0035639)		
		protein binding (GO:0005515)		
		nucleic acid binding (GO:0003676)		
		ion binding (GO:0043167)		
		protein serine/threonine kinase activity (GO:0004674)		
		organic cyclic compound binding (GO:0097159)		
		metal ion transmembrane transporter activity (GO:0046873)		
		inorganic molecular entity transmembrane transporter activity (GO:0015318)		
		translation regulator activity (GO:0045182)		
		metallopeptidase activity (GO:0008237)		
ND BP	6	growth factor binding (GO:0019838)		
		sequence-specific double-stranded DNA binding (GO:1990837)		
		disulfide oxidoreductase activity (GO:0015036)		
		transcription regulatory region sequence-specific DNA binding (GO:0000976)		
		molecular function regulator (GO:0098772)		
		collagen binding (GO:0005518)		
HFD BP	11	transferase activity, transferring phosphorus-containing groups (GO:0016772)		
		active transmembrane transporter activity (GO:0022804)		
		heat shock protein binding (GO:0031072)		
		translation initiation factor binding (GO:0031369)		
		ATP binding (GO:0005524)		
		protein kinase activity (GO:0004672)		
		phosphotransferase activity, alcohol group as acceptor (GO:0016773)		
		2-oxoglutarate-dependent dioxygenase activity (GO:0016706)		
		kinase activity (GO:0016301)		
		monovalent inorganic cation transmembrane transporter activity (GO:0015077)		
		transferase activity (GO:0016740)		
