# Supplementary material for: Obesity induced by high-fat diet is associated with critical changes in biological and molecular functions of mesenchymal stromal cells present in visceral adipose tissue
Source: Aging (Albany NY). 2020 Dec 27;12(24):24894–913. doi: 10.18632/aging.202423 (PMC7803587; doi:10.18632/aging.202423)
Supplement: Supplementary File 3 [file aging-12-202423-s003.pdf]

Supplementary File 3. Reactome, canonical pathways, upstream regulators, and Venn analysis.

Names	total	elements
DIO ND	239	PTEN Regulation
		p53-Dependent G1 DNA Damage Response
		Chaperonin-mediated protein folding
		SUMOylation of DNA replication proteins
		Vpu mediated degradation of CD4
		Nonsense Mediated Decay (NMD) independent of the Exon Junction Complex (EJC)
		Processing of Capped Intron-Containing Pre-mRNA
		Nuclear import of Rev protein
		Macroautophagy
		HSF1 activation
		Clathrin-mediated endocytosis
		Switching of origins to a post-replicative state
		Late Phase of HIV Life Cycle
		Response of EIF2AK4 (GCN2) to amino acid deficiency
		ISG15 antiviral mechanism
		Dectin-1 mediated noncanonical NF-kB signaling
		Translation
		COPI-independent Golgi-to-ER retrograde traffic
		G2/M Transition
		Interactions of Rev with host cellular proteins
		Autodegradation of the E3 ubiquitin ligase COP1
		Vpr-mediated nuclear import of PICs
		NIK-->noncanonical NF-kB signaling
		RNA Polymerase II Transcription Termination
		S Phase
		Transport of Mature mRNA derived from an Intron-Containing Transcript
		Cyclin A:Cdk2-associated events at S phase entry
		Nuclear Pore Complex (NPC) Disassembly
		Cellular responses to stress
		Folding of actin by CCT/TriC
		Regulation of APC/C activators between G1/S and early anaphase
		Attenuation phase
		Signaling by NOTCH4
		APC/C-mediated degradation of cell cycle proteins
		Cdc20:Phospho-APC/C mediated degradation of Cyclin A
		Activation of APC/C and APC/C:Cdc20 mediated degradation of mitotic proteins
		Hedgehog 'off' state
		UCH proteinases
		p53-Independent DNA Damage Response
		Cellular response to heat stress
		Nonsense Mediated Decay (NMD) enhanced by the Exon Junction Complex (EJC)
		Neutrophil degranulation
		Gene and protein expression by JAK-STAT signaling after Interleukin-12 stimulation
		Transport of the SLBP independent Mature mRNA
		Nuclear Envelope (NE) Reassembly
		Degradation of GLI2 by the proteasome
		Transport of Mature mRNAs Derived from Intronless Transcripts
		Ub-specific processing proteases
		Apoptosis induced DNA fragmentation
		Regulation of RUNX2 expression and activity
		Mitotic Prometaphase
		Translocation of SLC2A4 (GLUT4) to the plasma membrane
		RUNX1 regulates transcription of genes involved in differentiation of HSCs
		APC:Cdc20 mediated degradation of cell cycle proteins prior to satisfation of the cell cycle checkpoint
		Metabolism of RNA
		Membrane Trafficking
		p53-Independent G1/S DNA damage checkpoint
		Degradation of beta-catenin by the destruction complex
		Regulation of Apoptosis
		Glycolysis
		Apoptosis
		Vesicle-mediated transport
		Interactions of Vpr with host cellular proteins
		Transport of Ribonucleoproteins into the Host Nucleus
		ABC-family proteins mediated transport
		SUMOylation of SUMOylation proteins
		APC/C:Cdc20 mediated degradation of mitotic proteins
		Signaling by Hedgehog
		Cross-presentation of soluble exogenous antigens (endosomes)
		CLEC7A (Dectin-1) signaling
		Hedgehog 'on' state
		Transport to the Golgi and subsequent modification
		p53-Dependent G1/S DNA damage checkpoint
		TCR signaling
		CDT1 association with the CDC6:ORC:origin complex
		Hh mutants that don't undergo autocatalytic processing are degraded by ERAD
		Assembly of the pre-replicative complex
		APC/C:Cdh1 mediated degradation of Cdc20 and other APC/C:Cdh1 targeted proteins in late mitosis/early G1
		Aggrephagy
		Hh mutants abrogate ligand secretion
		DNA Replication Pre-Initiation
		Regulation of ornithine decarboxylase (ODC)
		XBP1(S) activates chaperone genes
		Defective CFTR causes cystic fibrosis
		Mitochondrial protein import
		SUMOylation of chromatin organization proteins
		Autophagy
		Metabolism of polyamines
		Orc1 removal from chromatin
		M Phase
		MHC class II antigen presentation
		mRNA Splicing - Major Pathway
		Metabolism of proteins
		Regulation of PTEN stability and activity
		trans-Golgi Network Vesicle Budding
		Major pathway of rRNA processing in the nucleolus and cytosol
		FGFR2 alternative splicing
		Separation of Sister Chromatids
		Vif-mediated degradation of APOBEC3G
		Selenocysteine synthesis
		Negative regulation of NOTCH4 signaling
		Prefoldin mediated transfer of substrate to CCT/TriC
		Host Interactions of HIV factors
		RHO GTPases Activate WASPs and WAVEs
		Viral Messenger RNA Synthesis
		Transcriptional regulation by RUNX3
		rRNA processing
		Downstream TCR signaling
		Unfolded Protein Response (UPR)
		DNA Replication
		Complex I biogenesis
		Eukaryotic Translation Termination
		Nervous system development
		Interleukin-1 signaling
		Antiviral mechanism by IFN-stimulated genes
		Regulation of HSF1-mediated heat shock response
		Cytosolic tRNA aminoacylation
		G1/S DNA Damage Checkpoints
		Activation of the mRNA upon binding of the cap-binding complex and eIFs, and subsequent binding to 43S
		Rev-mediated nuclear export of HIV RNA
		ER to Golgi Anterograde Transport
		Stabilization of p53
		Eukaryotic Translation Elongation
		CDK-mediated phosphorylation and removal of Cdc6
		PCP/CE pathway
		Mitotic G2-G2/M phases
		Axon guidance
		Post-chaperonin tubulin folding pathway
		HSF1-dependent transactivation
		Degradation of AXIN
		Regulation of mitotic cell cycle
		The citric acid (TCA) cycle and respiratory electron transport
		Nuclear Envelope Breakdown
		Programmed Cell Death
		NS1 Mediated Effects on Host Pathways
		RHO GTPases Activate Formins
		HIV Life Cycle
		Influenza Infection
		L13a-mediated translational silencing of Ceruloplasmin expression
		Beta-catenin independent WNT signaling
		Peptide chain elongation
		GLI3 is processed to GLI3R by the proteasome
		Metabolism of non-coding RNA
		Translation initiation complex formation
		Viral mRNA Translation
		SCF(Skp2)-mediated degradation of p27/p21
		Activation of NF-kappaB in B cells
		snRNP Assembly
		Mitotic Prophase
		Oxygen-dependent proline hydroxylation of Hypoxia-inducible Factor Alpha
		Cristae formation
		COPI-mediated anterograde transport
		Protein folding
		Signaling by ROBO receptors
		Selenoamino acid metabolism
		Eukaryotic Translation Initiation
		NEP/NS2 Interacts with the Cellular Export Machinery
		Cap-dependent Translation Initiation
		Transport of the SLBP Dependant Mature mRNA
		ABC transporter disorders
		IRE1alpha activates chaperones
		Mitotic Anaphase
		Protein localization
		SUMOylation of RNA binding proteins
		Golgi Associated Vesicle Biogenesis
		Selective autophagy
		Regulation of Glucokinase by Glucokinase Regulatory Protein
		Cell-extracellular matrix interactions
		HIV Infection
		HSP90 chaperone cycle for steroid hormone receptors (SHR)
		Signaling by Interleukins
		Interleukin-12 family signaling
		G1/S Transition
		APC/C:Cdc20 mediated degradation of Securin
		Transcriptional regulation by small RNAs
		Ubiquitin-dependent degradation of Cyclin D
		SRP-dependent cotranslational protein targeting to membrane
		Golgi-to-ER retrograde transport
		Postmitotic nuclear pore complex (NPC) reformation
		L1CAM interactions
		Cell Cycle Checkpoints
		Asymmetric localization of PCP proteins
		G2/M Checkpoints
		Respiratory electron transport
		Cellular responses to external stimuli
		Degradation of DVL
		Ribosomal scanning and start codon recognition
		Glucose metabolism
		Hedgehog ligand biogenesis
		RHO GTPase Effectors
		Transport of Mature Transcript to Cytoplasm
		Autodegradation of Cdh1 by Cdh1:APC/C
		Synthesis of DNA
		Respiratory electron transport, ATP synthesis by chemiosmotic coupling, and heat production by uncoupling proteins.
		mRNA Splicing - Minor Pathway
		Cellular response to hypoxia
		Downstream signaling events of B Cell Receptor (BCR)
		COPI-dependent Golgi-to-ER retrograde traffic
		rRNA processing in the nucleus and cytosol
		Transcriptional regulation by RUNX2
		Metabolism of amino acids and derivatives
		Export of Viral Ribonucleoproteins from Nucleus
		TNFR2 non-canonical NF-kB pathway
		Interleukin-12 signaling
		Deubiquitination
		Formation of the ternary complex, and subsequently, the 43S complex
		Regulation of mRNA stability by proteins that bind AU-rich elements
		Influenza Viral RNA Transcription and Replication
		Asparagine N-linked glycosylation
		Cyclin E associated events during G1/S transition
		Gene Silencing by RNA
		MAPK6/MAPK4 signaling
		Regulation of activated PAK-2p34 by proteasome mediated degradation
		Mitotic G1 phase and G1/S transition
		Cell Cycle
		Mitotic Metaphase and Anaphase
		Cooperation of Prefoldin and TriC/CCT in actin and tubulin folding
		mRNA Splicing
		Formation of a pool of free 40S subunits
		Ubiquitin Mediated Degradation of Phosphorylated Cdc25A
		Intra-Golgi and retrograde Golgi-to-ER traffic
		Regulation of RUNX3 expression and activity
		mRNA 3'-end processing
		Formation of tubulin folding intermediates by CCT/TriC
		tRNA processing in the nucleus
		Transport of Mature mRNA Derived from an Intronless Transcript
		GTP hydrolysis and joining of the 60S ribosomal subunit
		SUMOylation of ubiquitinylation proteins
		Degradation of GLI1 by the proteasome
		Defective TPR may confer susceptibility towards thyroid papillary carcinoma (TPC)
		Nonsense-Mediated Decay (NMD)
		The role of GTSE1 in G2/M progression after G2 checkpoint
		SCF-beta-TrCP mediated degradation of Emi1
		FBXL7 down-regulates AURKA during mitotic entry and in early mitosis
		Recycling pathway of L1
		Cell Cycle, Mitotic
		AUF1 (hnRNP D0) binds and destabilizes mRNA
		Regulation of RAS by GAPs
		Regulation of expression of SLITs and ROBOs
ND	19	Resolution of Sister Chromatid Cohesion
		Platelet degranulation
		Signal transduction by L1
		COPII-mediated vesicle transport
		Mitochondrial translation elongation
		Response to elevated platelet cytosolic Ca2+
		Synthesis of active ubiquitin: roles of E1 and E2 enzymes
		Formation of TC-NER Pre-Incision Complex
		RHO GTPases activate KTN1
		RHO GTPases activate CIT
		Amplification of signal from unattached kinetochores via a MAD2 inhibitory signal
		Signaling by Rho GTPases
		Amplification of signal from the kinetochores
		Neddylation
		RHO GTPases Activate ROCKs
		EML4 and NUDC in mitotic spindle formation
		Mitochondrial translation
		Mitochondrial translation initiation
		Mitochondrial translation termination
DIO	17	AURKA Activation by TPX2
		SUMOylation of DNA damage response and repair proteins
		Intracellular signaling by second messengers
		Apoptotic execution phase
		Cargo recognition for clathrin-mediated endocytosis
		Infectious disease
		Signaling by WNT
		MAPK family signaling cascades
		Processing of Capped Intronless Pre-mRNA
		Interleukin-1 family signaling
		tRNA Aminoacylation
		Signaling by MET
		Signaling by FGFR2
		Signaling by BRAF and RAF fusions
		TCF dependent signaling in response to WNT
		Regulation of PLK1 Activity at G2/M Transition
		PIP3 activates AKT signaling

Names	total	elements
DIO ND	232	PTEN Regulation
		p53-Dependent G1 DNA Damage Response
		Chaperonin-mediated protein folding
		Transcriptional regulation by RUNX1
		Vpu mediated degradation of CD4
		Nonsense Mediated Decay (NMD) independent of the Exon Junction Complex (EJC)
		Antigen processing-Cross presentation
		Extracellular matrix organization
		MET promotes cell motility
		MET activates PTK2 signaling
		Switching of origins to a post-replicative state
		Collagen formation
		Innate Immune System
		Response of EIF2AK4 (GCN2) to amino acid deficiency
		Dectin-1 mediated noncanonical NF-kB signaling
		Translation
		COPI-independent Golgi-to-ER retrograde traffic
		G2/M Transition
		Platelet degranulation
		Autodegradation of the E3 ubiquitin ligase COP1
		NIK-->noncanonical NF-kB signaling
		Smooth Muscle Contraction
		S Phase
		Cyclin A:Cdk2-associated events at S phase entry
		Non-integrin membrane-ECM interactions
		Cellular responses to stress
		Folding of actin by CCT/TriC
		Regulation of APC/C activators between G1/S and early anaphase
		Signaling by NOTCH4
		APC/C-mediated degradation of cell cycle proteins
		Cdc20:Phospho-APC/C mediated degradation of Cyclin A
		Activation of APC/C and APC/C:Cdc20 mediated degradation of mitotic proteins
		Hedgehog 'off' state
		UCH proteinases
		Syndecan interactions
		p53-Independent DNA Damage Response
		RHO GTPases activate PAKs
		Signaling by NOTCH
		Nonsense Mediated Decay (NMD) enhanced by the Exon Junction Complex (EJC)
		Neutrophil degranulation
		EPH-Ephrin signaling
		Gene and protein expression by JAK-STAT signaling after Interleukin-12 stimulation
		Nuclear Envelope (NE) Reassembly
		Degradation of GLI2 by the proteasome
		Fc epsilon receptor (FCERI) signaling
		Ub-specific processing proteases
		Regulation of RUNX2 expression and activity
		Response to elevated platelet cytosolic Ca2+
		Translocation of SLC2A4 (GLUT4) to the plasma membrane
		RUNX1 regulates transcription of genes involved in differentiation of HSCs
		APC:Cdc20 mediated degradation of cell cycle proteins prior to satisfation of the cell cycle checkpoint
		Metabolism of RNA
		p53-Independent G1/S DNA damage checkpoint
		Degradation of beta-catenin by the destruction complex
		Regulation of Apoptosis
		Apoptosis
		Disease
		Post-translational protein modification
		ABC-family proteins mediated transport
		APC/C:Cdc20 mediated degradation of mitotic proteins
		Collagen biosynthesis and modifying enzymes
		Signaling by Hedgehog
		Cross-presentation of soluble exogenous antigens (endosomes)
		CLEC7A (Dectin-1) signaling
		Hedgehog 'on' state
		p53-Dependent G1/S DNA damage checkpoint
		TCR signaling
		CDT1 association with the CDC6:ORC:origin complex
		Cytokine Signaling in Immune system
		Signaling by the B Cell Receptor (BCR)
		Hh mutants that don't undergo autocatalytic processing are degraded by ERAD
		Assembly of the pre-replicative complex
		ER-Phagosome pathway
		APC/C:Cdh1 mediated degradation of Cdc20 and other APC/C:Cdh1 targeted proteins in late mitosis/early G1
		Infectious disease
		Post-translational protein phosphorylation
		Aggrephagy
		Hh mutants abrogate ligand secretion
		DNA Replication Pre-Initiation
		XBP1(S) activates chaperone genes
		Regulation of ornithine decarboxylase (ODC)
		Defective CFTR causes cystic fibrosis
		Antigen processing: Ubiquitination & Proteasome degradation
		Signaling by WNT
		Orc1 removal from chromatin
		Metabolism of polyamines
		Assembly of collagen fibrils and other multimeric structures
		MAPK family signaling cascades
		M Phase
		MHC class II antigen presentation
		Metabolism of proteins
		Regulation of PTEN stability and activity
		Transport of connexons to the plasma membrane
		Major pathway of rRNA processing in the nucleolus and cytosol
		Developmental Biology
		Vif-mediated degradation of APOBEC3G
		Separation of Sister Chromatids
		Interleukin-1 family signaling
		Selenocysteine synthesis
		Negative regulation of NOTCH4 signaling
		Prefoldin mediated transfer of substrate to CCT/TriC
		Host Interactions of HIV factors
		C-type lectin receptors (CLRs)
		RHO GTPases Activate WASPs and WAVEs
		Transcriptional regulation by RUNX3
		Regulation of Insulin-like Growth Factor (IGF) transport and uptake by Insulin-like Growth Factor Binding Proteins (IGFBPs)
		rRNA processing
		NCAM1 interactions
		Downstream TCR signaling
		Unfolded Protein Response (UPR)
		Microtubule-dependent trafficking of connexons from Golgi to the plasma membrane
		DNA Replication
		Eukaryotic Translation Termination
		Elastic fibre formation
		Nervous system development
		Interleukin-1 signaling
		Cytosolic tRNA aminoacylation
		G1/S DNA Damage Checkpoints
		Activation of the mRNA upon binding of the cap-binding complex and eIFs, and subsequent binding to 43S
		Recruitment of NuMA to mitotic centrosomes
		Degradation of the extracellular matrix
		Stabilization of p53
		Eukaryotic Translation Elongation
		CDK-mediated phosphorylation and removal of Cdc6
		PCP/CE pathway
		Mitotic G2-G2/M phases
		Axon guidance
		Post-chaperonin tubulin folding pathway
		tRNA Aminoacylation
		Degradation of AXIN
		Regulation of mitotic cell cycle
		Platelet activation, signaling and aggregation
		Signaling by Rho GTPases
		Programmed Cell Death
		Semaphorin interactions
		RHO GTPases Activate Formins
		Interleukin-4 and Interleukin-13 signaling
		Influenza Infection
		L13a-mediated translational silencing of Ceruloplasmin expression
		Beta-catenin independent WNT signaling
		Peptide chain elongation
		RAF/MAP kinase cascade
		GLI3 is processed to GLI3R by the proteasome
		Signaling by Receptor Tyrosine Kinases
		Translation initiation complex formation
		Viral mRNA Translation
		SCF(Skp2)-mediated degradation of p27/p21
		Laminin interactions
		Activation of NF-kappaB in B cells
		Anchoring fibril formation
		MAPK1/MAPK3 signaling
		FLT3 Signaling
		Oxygen-dependent proline hydroxylation of Hypoxia-inducible Factor Alpha
		Dissolution of Fibrin Clot
		Protein folding
		COPI-mediated anterograde transport
		Signaling by ROBO receptors
		Selenoamino acid metabolism
		Eukaryotic Translation Initiation
		Cap-dependent Translation Initiation
		RHO GTPases activate IQGAPs
		ABC transporter disorders
		IRE1alpha activates chaperones
		Mitotic Anaphase
		Selective autophagy
		Cell-extracellular matrix interactions
		HIV Infection
		HSP90 chaperone cycle for steroid hormone receptors (SHR)
		Signaling by Interleukins
		Interleukin-12 family signaling
		G1/S Transition
		APC/C:Cdc20 mediated degradation of Securin
		Ubiquitin-dependent degradation of Cyclin D
		SRP-dependent cotranslational protein targeting to membrane
		Golgi-to-ER retrograde transport
		L1CAM interactions
		Cell Cycle Checkpoints
		Neddylation
		Asymmetric localization of PCP proteins
		G2/M Checkpoints
		Cellular responses to external stimuli
		Degradation of DVL
		Ribosomal scanning and start codon recognition
		Hedgehog ligand biogenesis
		RHO GTPase Effectors
		Autodegradation of Cdh1 by Cdh1:APC/C
		Synthesis of DNA
		Cellular response to hypoxia
		Crosslinking of collagen fibrils
		Association of TriC/CCT with target proteins during biosynthesis
		Downstream signaling events of B Cell Receptor (BCR)
		rRNA processing in the nucleus and cytosol
		Transcriptional regulation by RUNX2
		Metabolism of amino acids and derivatives
		TNFR2 non-canonical NF-kB pathway
		Molecules associated with elastic fibres
		Interleukin-12 signaling
		Deubiquitination
		Formation of the ternary complex, and subsequently, the 43S complex
		Influenza Viral RNA Transcription and Replication
		Regulation of mRNA stability by proteins that bind AU-rich elements
		EPHB-mediated forward signaling
		Cyclin E associated events during G1/S transition
		Collagen degradation
		ECM proteoglycans
		TCF dependent signaling in response to WNT
		MAPK6/MAPK4 signaling
		Regulation of activated PAK-2p34 by proteasome mediated degradation
		Mitotic G1 phase and G1/S transition
		FCERI mediated NF-kB activation
		Cell Cycle
		Mitotic Metaphase and Anaphase
		Cooperation of Prefoldin and TriC/CCT in actin and tubulin folding
		Formation of a pool of free 40S subunits
		Ubiquitin Mediated Degradation of Phosphorylated Cdc25A
		Regulation of RUNX3 expression and activity
		Formation of tubulin folding intermediates by CCT/TriC
		Collagen chain trimerization
		GTP hydrolysis and joining of the 60S ribosomal subunit
		Degradation of GLI1 by the proteasome
		Nonsense-Mediated Decay (NMD)
		The role of GTSE1 in G2/M progression after G2 checkpoint
		SCF-beta-TrCP mediated degradation of Emi1
		FBXL7 down-regulates AURKA during mitotic entry and in early mitosis
		Recycling pathway of L1
		Cell Cycle, Mitotic
		AUF1 (hnRNP D0) binds and destabilizes mRNA
		Integrin cell surface interactions
		Regulation of RAS by GAPs
		Regulation of expression of SLITs and ROBOs
		Diseases associated with glycosaminoglycan metabolism
		Immune System
ND	8	Adaptive Immune System
		Hemostasis
		Class I MHC mediated antigen processing & presentation
		Chk1/Chk2(Cds1) mediated inactivation of Cyclin B:Cdk1 complex
		Detoxification of Reactive Oxygen Species
		Gluconeogenesis
		Activation of Matrix Metalloproteinases
		Activation of BAD and translocation to mitochondria
DIO	32	NCAM signaling for neurite out-growth
		Processing of Capped Intron-Containing Pre-mRNA
		HSF1 activation
		Chaperone Mediated Autophagy
		Defective B3GAT3 causes JDSSDHD
		Loss of Nlp from mitotic centrosomes
		Attenuation phase
		Cellular response to heat stress
		Defective EXT2 causes exostoses 2
		Signaling by PDGF
		Membrane Trafficking
		Advanced glycosylation endproduct receptor signaling
		Vesicle-mediated transport
		Transport to the Golgi and subsequent modification
		Antagonism of Activin by Follistatin
		mRNA Splicing - Major Pathway
		RHO GTPases activate CIT
		Regulation of HSF1-mediated heat shock response
		ER to Golgi Anterograde Transport
		HSF1-dependent transactivation
		Loss of proteins required for interphase microtubule organization from the centrosome
		Signaling by MET
		Neurodegenerative Diseases
		Defective B4GALT7 causes EDS, progeroid type
		Defective B3GALT6 causes EDSP2 and SEMDJL1
		RHO GTPases Activate ROCKs
		COPI-dependent Golgi-to-ER retrograde traffic
		mRNA Splicing
		Intra-Golgi and retrograde Golgi-to-ER traffic
		Regulation of PLK1 Activity at G2/M Transition
		Deregulated CDK5 triggers multiple neurodegenerative pathways in Alzheimer's disease models
		Defective EXT1 causes exostoses 1, TRPS2 and CHDS

Names	total	elements
DIO ND	269	Reelin Signaling in Neurons
		Molecular Mechanisms of Cancer
		Dopamine Receptor Signaling
		fMLP Signaling in Neutrophils
		Xenobiotic Metabolism General Signaling Pathway
		UVA-Induced MAPK Signaling
		Role of NFAT in Regulation of the Immune Response
		Methionine Degradation I (to Homocysteine)
		LPS-stimulated MAPK Signaling
		Glycogen Degradation III
		Cardiac Hypertrophy Signaling
		Insulin Secretion Signaling Pathway
		IL-22 Signaling
		Signaling by Rho Family GTPases
		Neurotrophin/TRK Signaling
		Role of Tissue Factor in Cancer
		Fatty Acid Œ±-oxidation
		Senescence Pathway
		Role of NFAT in Cardiac Hypertrophy
		Ephrin Receptor Signaling
		DNA Methylation and Transcriptional Repression Signaling
		ErbB Signaling
		Melanocyte Development and Pigmentation Signaling
		Aspartate Degradation II
		Cancer Drug Resistance By Drug Efflux
		GNRH Signaling
		Isoleucine Degradation I
		Arginine Degradation VI (Arginase 2 Pathway)
		Adrenomedullin signaling pathway
		CNTF Signaling
		Unfolded protein response
		BMP signaling pathway
		Endocannabinoid Developing Neuron Pathway
		Mechanisms of Viral Exit from Host Cells
		Proline Biosynthesis II (from Arginine)
		Acetyl-CoA Biosynthesis I (Pyruvate Dehydrogenase Complex)
		Estrogen-Dependent Breast Cancer Signaling
		Apelin Adipocyte Signaling Pathway
		Glucose and Glucose-1-phosphate Degradation
		PFKFB4 Signaling Pathway
		Leukocyte Extravasation Signaling
		IL-6 Signaling
		Endometrial Cancer Signaling
		Regulation of IL-2 Expression in Activated and Anergic T Lymphocytes
		Remodeling of Epithelial Adherens Junctions
		Caveolar-mediated Endocytosis Signaling
		CTLA4 Signaling in Cytotoxic T Lymphocytes
		mTOR Signaling
		Telomerase Signaling
		UVC-Induced MAPK Signaling
		Neuregulin Signaling
		Pentose Phosphate Pathway (Oxidative Branch)
		Ephrin B Signaling
		RAN Signaling
		Xenobiotic Metabolism CAR Signaling Pathway
		Hypoxia Signaling in the Cardiovascular System
		Superpathway of Inositol Phosphate Compounds
		Ethanol Degradation IV
		GP6 Signaling Pathway
		Non-Small Cell Lung Cancer Signaling
		HIF1Œ± Signaling
		Role of PKR in Interferon Induction and Antiviral Response
		Prostate Cancer Signaling
		Synaptic Long Term Potentiation
		Acute Myeloid Leukemia Signaling
		Ethanol Degradation II
		Hepatic Fibrosis Signaling Pathway
		Histamine Degradation
		Leucine Degradation I
		RhoGDI Signaling
		PAK Signaling
		Synaptogenesis Signaling Pathway
		GŒ±12/13 Signaling
		Proline Biosynthesis I
		ErbB2-ErbB3 Signaling
		Salvage Pathways of Pyrimidine Ribonucleotides
		tRNA Charging
		Autophagy
		Tight Junction Signaling
		Huntington's Disease Signaling
		Colanic Acid Building Blocks Biosynthesis
		Opioid Signaling Pathway
		Xenobiotic Metabolism Signaling
		CCR3 Signaling in Eosinophils
		Inhibition of ARE-Mediated mRNA Degradation Pathway
		eNOS Signaling
		Prolactin Signaling
		PPARŒ±/RXRŒ± Activation
		Agrin Interactions at Neuromuscular Junction
		PDGF Signaling
		Ceramide Signaling
		Valine Degradation I
		Glycogen Biosynthesis II (from UDP-D-Glucose)
		Oxidative Ethanol Degradation III
		Tec Kinase Signaling
		VEGF Signaling
		Cellular Effects of Sildenafil (Viagra)
		Virus Entry via Endocytic Pathways
		Thioredoxin Pathway
		p70S6K Signaling
		ERK/MAPK Signaling
		Regulation of Actin-based Motility by Rho
		Mitochondrial Dysfunction
		Nitric Oxide Signaling in the Cardiovascular System
		Oncostatin M Signaling
		Aryl Hydrocarbon Receptor Signaling
		Aldosterone Signaling in Epithelial Cells
		D-myo-inositol (1,4,5,6)-Tetrakisphosphate Biosynthesis
		Protein Ubiquitination Pathway
		Tetrahydrofolate Salvage from 5,10-methenyltetrahydrofolate
		Putrescine Degradation III
		Macropinocytosis Signaling
		Superpathway of Geranylgeranyldiphosphate Biosynthesis I (via Mevalonate)
		Erythropoietin Signaling
		Thrombin Signaling
		D-myo-inositol (3,4,5,6)-tetrakisphosphate Biosynthesis
		NF-Œ∫B Activation by Viruses
		Integrin Signaling
		Superpathway of Methionine Degradation
		Œ≥-linolenate Biosynthesis II (Animals)
		HGF Signaling
		PI3K/AKT Signaling
		Glutathione Biosynthesis
		GDP-mannose Biosynthesis
		Glioma Invasiveness Signaling
		Lipid Antigen Presentation by CD1
		EGF Signaling
		IL-17 Signaling
		Axonal Guidance Signaling
		GM-CSF Signaling
		Glutaryl-CoA Degradation
		PPAR Signaling
		SPINK1 General Cancer Pathway
		IL-2 Signaling
		Semaphorin Signaling in Neurons
		Epithelial Adherens Junction Signaling
		Role of JAK1 and JAK3 in Œ≥c Cytokine Signaling
		Mevalonate Pathway I
		AMPK Signaling
		NER Pathway
		Cdc42 Signaling
		Antiproliferative Role of Somatostatin Receptor 2
		Natural Killer Cell Signaling
		GDNF Family Ligand-Receptor Interactions
		ERK5 Signaling
		Cysteine Biosynthesis III (mammalia)
		Gap Junction Signaling
		G Beta Gamma Signaling
		RhoA Signaling
		Glioblastoma Multiforme Signaling
		Superpathway of Serine and Glycine Biosynthesis I
		IL-8 Signaling
		Renin-Angiotensin Signaling
		Production of Nitric Oxide and Reactive Oxygen Species in Macrophages
		IL-3 Signaling
		Coronavirus Pathogenesis Pathway
		GŒ±q Signaling
		Role of MAPK Signaling in the Pathogenesis of Influenza
		NGF Signaling
		ILK Signaling
		Paxillin Signaling
		Glioma Signaling
		Colorectal Cancer Metastasis Signaling
		Heme Biosynthesis from Uroporphyrinogen-III I
		Gluconeogenesis I
		GDP-glucose Biosynthesis
		3-phosphoinositide Degradation
		PEDF Signaling
		Semaphorin Neuronal Repulsive Signaling Pathway
		Thrombopoietin Signaling
		Melatonin Signaling
		SAPK/JNK Signaling
		UDP-N-acetyl-D-glucosamine Biosynthesis II
		Granzyme A Signaling
		Purine Nucleotides De Novo Biosynthesis II
		Chronic Myeloid Leukemia Signaling
		Glutathione Redox Reactions II
		Œ±-Adrenergic Signaling
		Renal Cell Carcinoma Signaling
		Hereditary Breast Cancer Signaling
		NADH Repair
		PTEN Signaling
		Angiopoietin Signaling
		Fatty Acid Œ≤-oxidation I
		JAK/Stat Signaling
		TCA Cycle II (Eukaryotic)
		Clathrin-mediated Endocytosis Signaling
		Role of JAK family kinases in IL-6-type Cytokine Signaling
		BAG2 Signaling Pathway
		Dopamine Degradation
		Phagosome Maturation
		UDP-N-acetyl-D-galactosamine Biosynthesis II
		Phenylethylamine Degradation I
		Chemokine Signaling
		Methylglyoxal Degradation III
		EIF2 Signaling
		Phospholipase C Signaling
		IL-15 Signaling
		Iron homeostasis signaling pathway
		Cleavage and Polyadenylation of Pre-mRNA
		CDK5 Signaling
		Actin Nucleation by ARP-WASP Complex
		FLT3 Signaling in Hematopoietic Progenitor Cells
		14-3-3-mediated Signaling
		Inosine-5'-phosphate Biosynthesis II
		Glycolysis I
		Fc Epsilon RI Signaling
		Ketogenesis
		Systemic Lupus Erythematosus Signaling
		Sertoli Cell-Sertoli Cell Junction Signaling
		UVB-Induced MAPK Signaling
		IL-1 Signaling
		Noradrenaline and Adrenaline Degradation
		ErbB4 Signaling
		Necroptosis Signaling Pathway
		Amyloid Processing
		Heme Biosynthesis II
		Arginine Biosynthesis IV
		Superpathway of Cholesterol Biosynthesis
		CXCR4 Signaling
		Xenobiotic Metabolism PXR Signaling Pathway
		Sirtuin Signaling Pathway
		Regulation of Cellular Mechanics by Calpain Protease
		ATM Signaling
		Oxidative Phosphorylation
		Germ Cell-Sertoli Cell Junction Signaling
		Pentose Phosphate Pathway
		Pyridoxal 5'-phosphate Salvage Pathway
		Apelin Endothelial Signaling Pathway
		P2Y Purigenic Receptor Signaling Pathway
		Retinoate Biosynthesis I
		Cholecystokinin/Gastrin-mediated Signaling
		B Cell Receptor Signaling
		Tryptophan Degradation X (Mammalian, via Tryptamine)
		Protein Kinase A Signaling
		RAR Activation
		Rac Signaling
		Apoptosis Signaling
		Actin Cytoskeleton Signaling
		HIPPO signaling
		Acute Phase Response Signaling
		Superoxide Radicals Degradation
		D-myo-inositol-5-phosphate Metabolism
		Estrogen Receptor Signaling
		Prostanoid Biosynthesis
		Insulin Receptor Signaling
		Tryptophan Degradation III (Eukaryotic)
		CREB Signaling in Neurons
		Pyrimidine Ribonucleotides De Novo Biosynthesis
		Dopamine-DARPP32 Feedback in cAMP Signaling
		Mouse Embryonic Stem Cell Pluripotency
		Spliceosomal Cycle
		Sumoylation Pathway
		FAK Signaling
		Xenobiotic Metabolism AHR Signaling Pathway
		Stearate Biosynthesis I (Animals)
		Androgen Signaling
		FcŒ≥ Receptor-mediated Phagocytosis in Macrophages and Monocytes
		Pancreatic Adenocarcinoma Signaling
		Ketolysis
		IGF-1 Signaling
		Heme Degradation
		Glycogen Degradation II
		Mitotic Roles of Polo-Like Kinase
		Glucocorticoid Receptor Signaling
		FAT10 Signaling Pathway
		Melanoma Signaling
		Regulation of eIF4 and p70S6K Signaling
		NRF2-mediated Oxidative Stress Response
ND	23	Hepatic Fibrosis / Hepatic Stellate Cell Activation
		Thyroid Cancer Signaling
		Cardiac Hypertrophy Signaling (Enhanced)
		Glutathione Redox Reactions I
		NF-Œ∫B Signaling
		Death Receptor Signaling
		2-oxobutanoate Degradation I
		Methylmalonyl Pathway
		FAT10 Cancer Signaling Pathway
		VEGF Family Ligand-Receptor Interactions
		Ovarian Cancer Signaling
		TGF-Œ≤ Signaling
		GŒ±i Signaling
		Synaptic Long Term Depression
		Leptin Signaling in Obesity
		PI3K Signaling in B Lymphocytes
		Regulation Of The Epithelial Mesenchymal Transition By Growth Factors Pathway
		Regulation of the Epithelial-Mesenchymal Transition Pathway
		Cardiac Œ≤-adrenergic Signaling
		HMGB1 Signaling
		Pyrimidine Deoxyribonucleotides De Novo Biosynthesis I
		HER-2 Signaling in Breast Cancer
		T Cell Receptor Signaling
DIO	12	CD40 Signaling
		Kinetochore Metaphase Signaling Pathway
		PRPP Biosynthesis I
		Apelin Liver Signaling Pathway
		Pyrimidine Ribonucleotides Interconversion
		3-phosphoinositide Biosynthesis
		Endocannabinoid Cancer Inhibition Pathway
		Apelin Cardiomyocyte Signaling Pathway
		4-1BB Signaling in T Lymphocytes
		Cell Cycle: G2/M DNA Damage Checkpoint Regulation
		Molybdenum Cofactor Biosynthesis
		Cell Cycle Control of Chromosomal Replication

Names	total	elements		
DIO ND	93	Reelin Signaling in Neurons		
		Sertoli Cell-Sertoli Cell Junction Signaling		
		Dopamine Receptor Signaling		
		Cdc42 Signaling		
		Intrinsic Prothrombin Activation Pathway		
		Gap Junction Signaling		
		Granulocyte Adhesion and Diapedesis		
		RhoA Signaling		
		RhoGDI Signaling		
		Glycogen Degradation III		
		Synaptogenesis Signaling Pathway		
		IL-8 Signaling		
		Coronavirus Pathogenesis Pathway		
		Arginine Biosynthesis IV		
		Signaling by Rho Family GTPases		
		Paxillin Signaling		
		ILK Signaling		
		Hepatic Fibrosis / Hepatic Stellate Cell Activation		
		Xenobiotic Metabolism PXR Signaling Pathway		
		Regulation of Cellular Mechanics by Calpain Protease		
		Ephrin Receptor Signaling		
		tRNA Charging		
		Aspartate Degradation II		
		Tight Junction Signaling		
		Huntington's Disease Signaling		
		Gluconeogenesis I		
		Germ Cell-Sertoli Cell Junction Signaling		
		CCR3 Signaling in Eosinophils		
		Pentose Phosphate Pathway		
		Semaphorin Neuronal Repulsive Signaling Pathway		
		Inhibition of ARE-Mediated mRNA Degradation Pathway		
		Agrin Interactions at Neuromuscular Junction		
		Purine Nucleotides De Novo Biosynthesis II		
		Protein Kinase A Signaling		
		5-aminoimidazole Ribonucleotide Biosynthesis I		
		VEGF Signaling		
		Unfolded protein response		
		Cellular Effects of Sildenafil (Viagra)		
		Formaldehyde Oxidation II (Glutathione-dependent)		
		Virus Entry via Endocytic Pathways		
		p70S6K Signaling		
		Mechanisms of Viral Exit from Host Cells		
		Rac Signaling		
		ERK/MAPK Signaling		
		Regulation of Actin-based Motility by Rho		
		Actin Cytoskeleton Signaling		
		TCA Cycle II (Eukaryotic)		
		HIPPO signaling		
		Clathrin-mediated Endocytosis Signaling		
		BAG2 Signaling Pathway		
		Aldosterone Signaling in Epithelial Cells		
		Protein Ubiquitination Pathway		
		Phagosome Maturation		
		Estrogen Receptor Signaling		
		Leukocyte Extravasation Signaling		
		Insulin Receptor Signaling		
		Remodeling of Epithelial Adherens Junctions		
		Caveolar-mediated Endocytosis Signaling		
		CTLA4 Signaling in Cytotoxic T Lymphocytes		
		mTOR Signaling		
		Agranulocyte Adhesion and Diapedesis		
		Dopamine-DARPP32 Feedback in cAMP Signaling		
		Thrombin Signaling		
		FAK Signaling		
		Xenobiotic Metabolism AHR Signaling Pathway		
		Pentose Phosphate Pathway (Oxidative Branch)		
		Integrin Signaling		
		Ephrin B Signaling		
		RAN Signaling		
		PI3K/AKT Signaling		
		Hypoxia Signaling in the Cardiovascular System		
		HIF1Œ± Signaling		
		GP6 Signaling Pathway		
		FcŒ≥ Receptor-mediated Phagocytosis in Macrophages and Monocytes		
		EIF2 Signaling		
		SPINK1 Pancreatic Cancer Pathway		
		Inhibition of Matrix Metalloproteases		
		IGF-1 Signaling		
		Role of PKR in Interferon Induction and Antiviral Response		
		Lipid Antigen Presentation by CD1		
		Glycogen Degradation II		
		Axonal Guidance Signaling		
		CDK5 Signaling		
		FAT10 Signaling Pathway		
		Cardiac Œ≤-adrenergic Signaling		
		Actin Nucleation by ARP-WASP Complex		
		14-3-3-mediated Signaling		
		Inosine-5'-phosphate Biosynthesis II		
		Semaphorin Signaling in Neurons		
		Epithelial Adherens Junction Signaling		
		Regulation of eIF4 and p70S6K Signaling		
		NRF2-mediated Oxidative Stress Response		
		Glycolysis I		
ND	20	fMLP Signaling in Neutrophils		
		Natural Killer Cell Signaling		
		Neuroprotective Role of THOP1 in Alzheimer's Disease		
		PAK Signaling		
		CXCR4 Signaling		
		3-phosphoinositide Degradation		
		Aryl Hydrocarbon Receptor Signaling		
		D-myo-inositol (1,4,5,6)-Tetrakisphosphate Biosynthesis		
		Acute Phase Response Signaling		
		D-myo-inositol-5-phosphate Metabolism		
		Chemokine Signaling		
		Complement System		
		D-myo-inositol (3,4,5,6)-tetrakisphosphate Biosynthesis		
		Neuregulin Signaling		
		Sucrose Degradation V (Mammalian)		
		Xenobiotic Metabolism CAR Signaling Pathway		
		LXR/RXR Activation		
		Osteoarthritis Pathway		
		Phospholipase C Signaling		
		Atherosclerosis Signaling		
DIO	9	Superpathway of Serine and Glycine Biosynthesis I		
		Sirtuin Signaling Pathway		
		Granzyme A Signaling		
		Oxidized GTP and dGTP Detoxification		
		Coagulation System		
		Superoxide Radicals Degradation		
		Spliceosomal Cycle		
		Superpathway of Methionine Degradation		
		Ephrin A Signaling		

Names	total	elements
DIO ND	384	VDR
		PPARD
		CD44
		ethoxyquin
		PAX7
		1,2-dithiol-3-thione
		ROCK1
		ZAP70
		ATF6
		Creb
		seocalcitol
		DCX
		EDN1
		PLG
		PPARGC1A
		galactose
		MEF2D
		CREB3L2
		N-propyl bromide
		CACNA1C
		Akt
		SOX7
		ILK
		BRD4
		NCOA3
		IGF1
		gemfibrozil
		MAPK14
		TGFB2
		PITX2
		AGN194204
		WNT1
		RAB1B
		isobutylmethylxanthine
		RAC1
		CCN2
		CCL5
		DPP-23
		TP53
		CD3
		decitabine
		D-glucose
		VBP1
		LINC01139
		homocysteine
		MITF
		dinoprost
		poly rI:rC-RNA
		PDGF BB
		STK11
		FLT1
		TNF
		inosine
		INSR
		TGFBR2
		RAPGEF1
		LCK
		E2f
		NCX-4040
		DDX5
		RAF1
		gentamicin C
		CAPN3
		ERG
		EIF4E
		HOTAIR
		5-hydroxytryptamine
		EGF
		USF2
		CCR2
		thioacetamide
		MMP3
		FGFR1
		1-methyl-4-phenyl-1,2,3,6-tetrahydropyridine
		dexamethasone
		SERPINH1
		H1f1
		butylated hydroxyanisol
		diethylnitrosamine
		PPARA
		cadmium
		SOX4
		KLF15
		CD300LF
		CD24
		TCF7L2
		Gm15807/Hmgn5
		Brd4
		PKM
		sodium arsenite
		TEAD1
		CALR
		LEP
		L-triiodothyronine
		SRC (family)
		LDL
		WR 1065
		MYCBP
		rhodioloside
		tert-butyl-hydroquinone
		SFPQ
		ELAVL1
		TGFB3
		Z-LLL-CHO
		sucrose
		PI3K (family)
		geldanamycin
		CAB39L
		LIMS2
		AKT1
		PRKCB
		JAK2
		PTPN3
		guanidinopropionic acid
		2-amino-1-methyl-6-phenylimidazo-[illegible]
		Rock
		MAP2K5
		Firre
		PRL
		Insulin
		ETV4
		NR1H3
		UTP
		ROCK2
		nitric oxide
		cisplatin
		MYCL
		SPP1
		LINC00842
		testosterone
		lomustine
		Ige
		F2
		cephaloridine
		OSM
		TFEB
		YAP1
		PI3K (complex)
		6-hydroxydopamine
		crocidolite asbestos
		IRAK3
		NCF1
		arsenite
		FGF7
		CTNNB1
		MLXIPL
		H1-6
		gentamicin
		MKNK1
		AKT2
		EGFR
		clofibrate
		IKBKB
		CBR-470-1
		SP1
		CD38
		aurothioglucose
		vancomycin
		VTN
		SYVN1
		allopurinol
		2-bromoethylamine
		bisphenol A
		EBF1
		ethionine
		diquat
		bortezomib
		phytohemagglutinin
		KDM8
		diethylmaleate
		arsenic
		HTT
		cardiotoxin
		PCGEM1
		UCP1
		SEMA7A
		IL15
		3,5-diiodothyronine
		ESR1
		ACLY
		ERBB2
		RLIM
		KITLG
		MAPK7
		ARNT
		LCN2
		rosiglitazone
		ELL2
		HBEGF
		ZBTB7B
		CCAR2
		PTK2
		FOS
		IFNG
		IPMK
		methotrexate
		FLI1
		17-alpha-ethinylestradiol
		CSF1
		RETN
		JUNB
		SMARCB1
		LLGL2
		IL4
		SREBF1
		bardoxolone
		CD28
		cis-urocanic acid
		HNRNPR
		MUC1
		pirinixic acid
		Collagen type I (complex)
		EPAS1
		bezafibrate
		carbon tetrachloride
		D-fructose
		mibolerone
		PDGF (family)
		TEAD3
		phosphate
		SPZ1
		RABL6
		FGF2
		genistein
		palmitic acid
		NORAD
		MAP2K4
		CH-223191
		Pdgf (complex)
		PFDN6
		acetaminophen
		LAMTOR1
		cadmium chloride
		SREBF2
		LIMS1
		VEGFA
		tretinoin
		mini-GAGR
		THRB
		CDH11
		triamterene
		LIN28A
		IL3
		GNA12
		sphingosine-1-phosphate
		TGFB1
		fenofibrate
		Cdc42
		ADORA2A
		EDNRA
		S100A6
		IGF1R
		CEBPB
		METTL3
		Tgf beta
		GAST
		Hif1
		EIF3M
		F10
		5-azacytidine
		HSF2
		beta-estradiol
		MYC
		F2R
		ESRRA
		ERN1
		PFDN5
		STAT4
		JUN
		nitrofurantoin
		BCL2L1
		KDM3A
		azetidyl-2-carboxylic acid
		TO-901317
		E2F1
		NSUN6
		NRG1
		ROR1
		estrogen
		FBXO32
		ciprofibrate
		PPARGC1B
		methapyrilene
		AR
		INHBB
		benzo(a)pyrene
		bleomycin
		TAZ
		Calcineurin A
		phenylbutazone
		MRTFA
		forskolin
		NFE2L1
		QKI
		methylprednisolone
		MAP2K3
		FOXF1
		elaidic acid
		methylnitrosourea
		SCAP
		AGTR2
		IL5
		AGT
		tunicamycin
		MAP3K1
		ERK
		E2F3
		Smad2/3/4
		MYCN
		HSP90B1
		EPO
		progesterone
		PPARG
		GNA13
		indomethacin
		MAFF
		ATF4
		hexachlorobenzene
		L-glutamic acid
		uranyl nitrate
		mono-(2-ethylhexyl)phthalate
		FOXO1
		lipopolysaccharide
		ASPSCR1-TFE3
		fenamic acid
		RHOA
		Hbb-b1
		TCR
		CD247
		IKBKE
		HIF1A
		PRKCA
		PLA2R1
		CD40LG
		HGF
		MRTFB
		SRF
		NR1D1
		EIF2AK3
		RET
		MASTL
		PTH
		cholic acid
		TCF4
		Srebp
		coal tar
		RB1
		SMYD1
		PDGFC
		prostaglandin J2
		lactacystin
		TP63
		APP
		EGR1
		(-)-norephedrine
		NFYA
		IGF2BP1
		thapsigargin
		Ins1
		dihydrotestosterone
		PNPLA2
		SRC
		HBA1/HBA2
		KNG1
		XBP1
		CASR
		MTPN
		metribolone
		FN1
		USF1
		hydrogen peroxide
		PGR
		PFDN2
		MIR143-145a
		ammonium chloride
		FGF10
		EIF2AK4
		ANGPT2
		Gm26313
		NFE2L2
		cinnamaldehyde
		NRF1
		cigarette smoke
		MYOD1
ND	70	Pro-inflammatory Cytokine
		TMPO
		IFI16
		desoxycorticosterone
		leupeptin
		AIFM1
		RAS
		WWTR1
		Pkc(s)
		PLA2G4A
		phenacetin
		ITGB6
		tetradecanoylphorbol acetate
		VIM
		NFASC
		mycophenolic acid
		S1PR2
		PIK3R1
		GLI1
		PTCD1
		RUVBL1
		Ras homolog
		BMP7
		TNFSF11
		MAP2K1
		THBS4
		IL1B
		FOXM1
		advanced glycation end-products
		CYP2E1
		tosedostat
		FGF21
		IL2
		ATM
		streptozocin
		PFKFB3
		farnesyl pyrophosphate
		SMARCA4
		F3
		BRD7
		NR1I2
		CXCL12
		anandamide
		lysophosphatidic acid
		SMAD4
		FUS
		RETNLB
		BML-284
		H2AX
		NOTCH3
		trypsin
		TIMP1
		AIF1
		aldesleukin
		ERK1/2
		AIMP2
		TWIST1
		JAG1
		aldosterone
		MMP1
		C11orf95-RELA
		Growth hormone
		platelet activating factor
		CXCL8
		1,4-bis[2-(3,5-dichloropyridyloxy)]benzene
		CRNDE
		deferoxamine
		ARNT2
		KLF6
		NR1I3
DIO	33	RPTOR
		BMP6
		PPP1R1B
		captopril
		RARA
		LOX
		CCN1
		NGF
		NEDD9
		NKX2-2-AS1
		beta-naphthoflavone
		NKX2-3
		reactive oxygen species
		3,4,5,3',4'-pentachlorobiphenyl
		Fus
		ITGB3
		CD3 group
		GNAQ
		lithium chloride
		GF 120918
		tetracycline
		iron
		HELLS
		DDX3X
		3-methylcholanthrene
		WBP2
		4-octyl itaconate
		bee venom
		triclosan
		isoquercitrin
		SOX2-OT
		CLDN7
		NUP107
