# Supplementary material for: Obesity induced by high-fat diet is associated with critical changes in biological and molecular functions of mesenchymal stromal cells present in visceral adipose tissue
Source: Aging (Albany NY). 2020 Dec 27;12(24):24894–913. doi: 10.18632/aging.202423 (PMC7803587; doi:10.18632/aging.202423)
Supplement: Supplementary File 4 [file aging-12-202423-s004.pdf]

Supplementary File 4. Differential quantitative analysis of cellular and secretome protein content.

HFD proteome vs ND proteome				
id	logFC	P Value	Protein_name	Upregulated in senescence
3442	1,8334452249	0,00269435727819142	Nek7	
1996	1,4691172261	0,00637964131571731	Smarcc2	
2043	1,273776346	0,00427104188496819	Thrap3	
840	1,1076583589	0,00168327364177417	Numa1	
1781	1,0478844769	0,0000699179905220381	Lxn	
1293	1,0276679473	0,0000154427661172483	Serpinb2	
70	1,0099596745	0,0000820693669692432	Crip1	
1214	0,9863040057	0,0000136239983333483	Thy1	
56	0,9134367494	0,00310207806841863	Kpna4	
1961	0,8704916762	0,00472139559712029	Rab31	
1492	0,8510793781	0,000216349151358706	Hist1h1a	
1126	0,8239814274	0,0046399200998519	Lsm2	
1093	0,8154065866	0,00958759922035111	Ddt;Gm20441	
1870	0,7706657627	0,000223676046784809	G6pdx	
1694	0,7605887669	0,00561272603045757	Snrpf	
768	0,7518840113	0,00252119798832818	Col12a1	
2772	0,7509053191	0,0030394986316694	Pex19	
1984	0,7450997055	0,00210462811418635	Puf60	
2455	0,7414553105	0,000942052399539338	Psmf1	
1664	0,7400835399	0,00395920046306446	Abce1	
838	0,7338546143	0,00018237317696891	Arf4	
1153	0,7310683576	0,000402226564670266	Psmb5	
2779	0,7239731892	0,000159671482182799	Bles03	
1982	0,7179381138	0,000728406655560883	Ube2z	
3379	0,7067613555	0,0039725245775567	Tmem43	
2142	0,702026471	0,0034957257296524	Pcolce	
1382	0,7019250479	0,0000636767390600667	Impdh2	
2370	0,6531585398	0,0000878864783831374	Sept9	
1418	0,6376365258	0,000864630547214175	Celf1	
2791	0,63522043	0,00910712256286143	Ddx39a;Ddx39	
1730	0,6248637051	0,00898567332494181	Gnb2	
559	0,6247005048	0,00134097624439539	Strn3	
3433	0,6141588346	0,00479236653969289	Snap29	
3638	0,6081528072	0,00286342657067619	Loxl3	
2967	0,597562709	0,00144775567021386	Sar1a	
517	0,5891440226	0,000564395997927024	Epb4.1l3	
527	0,5821554684	0,00133129032894773	Gabarap	
1593	0,577009692	0,00673844379708915	Adprh	
2765	0,57299942	0,000379420843294174	Tmx1	
3101	0,5588777771	0,000154144338524552	Txndc17	
1565	0,5459003577	0,000843785408692532	Slc25a5	
1769	0,5348017603	0,000586917515526476	Gnb2l1	
2215	0,5289606679	0,000762497834676732	Nqo1	
1705	0,5274155503	0,00444699392875865	Tsc22d1	
1172	0,5240038749	0,000852814660211086	Col5a1	
2360	0,5172932065	0,00292287610465646	Ahcyl1	
3266	0,5108509962	0,000874300666328217	Dlst	
2935	0,5102097708	0,00918314096784118	Blvrb	
1352	0,4960444443	0,000721022968436326	Rpl13a	
2442	0,4923333539	0,00477903574010604	Ehd2	
2387	0,4855651922	0,00106339539492248	Pelo	
1458	0,4594647629	0,00881971491573659	Fbl	
2047	0,4438004075	0,0018988656505307	Hspb6	
2841	0,437036317	0,00480992137517557	Rbmxl1	
1466	0,4362707353	0,00719566690578019	Rpl18	
1545	0,4356043072	0,00219280677630882	Ahcy	
1494	0,4243280795	0,00332912512209892	Hist1h1d	
1351	0,4240797174	0,00258600174132817	Gstp1;Gstp2	
3398	0,4162700876	0,00781009718902931	Cyb5r3	
939	0,4125739183	0,00242054968278032	Glg1	
2237	-0,4171430876	0,00394918246259585	Prdx6	
2083	-0,429846915	0,0019008182371437	P4ha2	
1548	-0,4301907935	0,00749028159740589	Atp6v1a	
1513	-0,4363438269	0,00359805020358121	Capzb	
2195	-0,4508875515	0,00294252603918987	Commd3	
2206	-0,4545562667	0,00899722546010801	Sqstm1	
2907	-0,468837533	0,00238261286124132	Acaa1a	
1370	-0,4688543909	0,00775316191203218	Ap1g1	
1572	-0,4734690934	0,000699111362203346	Ndufs6	
3348	-0,4807385148	0,000539778565527179	Cnn3	
619	-0,4819947838	0,00150926265893347	Sec11a	
2020	-0,4845952685	0,00406952094937324	Lrrfip1	
780	-0,4862770036	0,00174101842553539	Psap	
2987	-0,4887857995	0,00113832742191772	Dnajb11	
2192	-0,5089792259	0,00844836975491361	Vat1	
3568	-0,5136461564	0,0054250561689132	Acot9;Acot10	
2409	-0,5164030097	0,00174214346380595	Gns	
522	-0,5198421155	0,00340832318657111	Dusp3	
1222	-0,5219071793	0,0012696324148343	Mtatp8	
3491	-0,5233875349	0,00271207796449956	Ybx3	
51	-0,5400873095	0,00186917337911051	Cept1	
2297	-0,5438723788	0,00186985213467825	Calu	
217	-0,5530615732	0,000451209341334869	Prosc	
1567	-0,5531526643	0,00361461445470303	Kpna2	
1323	-0,5604245243	0,000629655160878608	Lgals3	
1003	-0,5622486582	0,00492516184842077	Lrrfip1	
1514	-0,5660053683	0,00113833613643509	Srprb	
2067	-0,5771492871	0,00328065798657825	Tns3	
1871	-0,5801912809	0,00456802460790942	Rbp1	
1328	-0,5866664457	0,000184960174876981	Ass1	
2262	-0,6002266143	0,00254940971504411	Nes	
2026	-0,6081667386	0,00531519399447231	Itga1	
2866	-0,6084007822	0,00495029756890851	Lims2	
948	-0,6205976315	0,000140759986005598	Tpm1	
1508	-0,6228967273	0,00410456352189489	Yap1	
2880	-0,6239431824	0,00558071003492162	Bag2	
2204	-0,6292146428	0,00012726790542773	Aga	
3056	-0,6332391974	0,0000975287533057685	Ftl1;Ftl2	
74	-0,6353548502	0,00713968664059502	Nexn	
1719	-0,6361377543	0,00134663820995571	Atp6v1b2	
2393	-0,6462689042	0,000466586223455754	Thbs1	
3198	-0,675181394	0,000220985156942475	Tsfm	
889	-0,7018615794	0,0000770663755695921	Myh11	
2481	-0,7178396396	0,00143208540946707	Nceh1	
1256	-0,7651591476	0,0000323679103997782	Fth1	
1084	-0,7908479654	0,0000372157667485105	Gsto1	
1577	-0,7953314645	0,00154683848411069	Mmp14	
1629	-0,8082426366	0,0000724778034086917	Tpm1	
926	-0,8120747646	0,000406068593760284	Ahnak2	
300	-0,8265892081	0,000184120720737982	Rai14	
1777	-0,8288741463	0,0066114052274039	Sbds	
1218	-0,8366228073	0,0095990944731073	Col4a1	
3533	-0,8416910155	0,00687816897689826	Cnpy2	
1078	-0,8482424424	0,00679520762468725	Degs1	
3122	-0,8698797125	0,00477874099518824	Mrps36	
3607	-0,8803308242	0,00030043417701372	Asah1	
874	-0,8815556632	0,00793399848353956	Asap1	
1317	-0,893105088	0,0020822713714668	Nme1	
3111	-0,9035832455	0,000214331558948957	Nop10	
1332	-0,9728773516	0,000543124996878448	Lamp2	
1246	-0,9849715771	0,000844096631315535	Col4a2	
1623	-1,0584390633	0,00768700956944666	Loxl2	
1316	-1,0774783281	0,000218977443961604	Thbd	
1345	-1,1772441259	0,00079581671571368	Cyr61	
2989	-1,2269581837	0,00567640533523095	Hibadh	
2734	-1,4234923211	0,00404860196117295	Ssbp1	
1283	-1,4353300895	0,000267419138330019	Lamp1	
3309	-1,440940833	0,00526860595315641	Iscu	
1434	-1,5001937981	0,00003436416096311	Des	
2756	-1,5874174324	0,00420531459819133	Luzp1	
1368	-1,5975717854	0,0000019182527285013	Tgm2	
94	-2,4597609205	0,000156308123227836	Specc1	
1000	-2,9584260772	0,00809088535576998	Serpine1	
1892	-3,4496484004	0,000000274314796637245	Ptgs2	

HFD proteome vs ND secretome				
id	logFC	P Value	Protein_name	Upregulated in senescence
739	2,4430875704	0000233604550899	Igfbp4	
338	1,7156946363	0000118164986369	Col6a3	
1009	1,6578769801	0010499787097263	Cttn	
138	1,3039468933	0002600756850402	Rpl10a	
1111	1,1005375142	0020557122703841	Ppp2r1a	
933	0,9400624304	0000004380158784	Thbs2	
811	0,9243342895	000333579436196	Hnrnpk	
520	0,9229528033	000013645790920	Col5a1	
1441	0,8524284598	000001361264223	Plec	
1341	0,8392556024	000032920730980	Hnrnpa0	
750	0,8064063348	0000011060241848	Lmna	
558	0,791382544	0000118339824921	Col3a1	
660	0,7777397525	000267522793177	Rdx	
1016	0,7151576134	000131213562763	Serpinb6;Serpinb6a	
535	0,7041810992	0,003565822959003	Rbm3	
752	0,6893529997	000096478859357	Hnrnpa1	
527	0,6491495327	000123712642445	Hnrnpa2b1	
819	0,614315932	0007373088507181	Rps8	
365	0,6128372157	000154423314879	Sept2	
467	0,6093337141	0012574794698781	Dpysl2	
372	0,5942341182	000000870465890	Ahnak	
956	0,59022227	0082405184188521	Hnrnpab	
1257	0,5861850855	0002056791718211	Cd248	
1059	0,5678391107	002017523956197	Sptbn1	
716	0,5631965404	000033743454633	Tkt	
411	0,5152591074	004980590819077	Hnrnpd	
567	0,5103727208	000027428085571	Ncl	
360	0,5084711089	001119699825402	Sept7	
1068	0,4997571198	000293437627676	Zyx	
637	0,4543034517	007272956817820	Tmsb4x	
581	0,4517376394	000012580919229	Col1a1	
392	0,4361955971	005588674164773	Lamb1	
586	0,4333681847	000033857059378	Hsp90ab1	
413	0,4324052824	000486620383282	Rps12	
523	0,4139587294	0003951553323821	Wdr1	
96	-0,416815187	000133026487511	Sparc	
673	-0,4460831088	0001006756851321	Lox	
1053	-0,4481338571	000339761840573	Postn	
701	-0,4802450865	000455980449396	Mif	
123	-0,5155660328	000416838362218	App	
1408	-0,5165073225	000160422889813	Palld	
223	-0,5371212934	002282943239690	Tpm1	
1067	-0,5408445879	0000926531456865	Vat1	
114	-0,5479371569	0,00939095042736	Fbln5	
566	-0,5706444813	000703227815937	Cck	
1473	-0,7064961396	000043194570144	Coro1c	
789	-0,7519233051	000187750654766	Loxl2	
642	-0,7673988833	003699426233060	Cst3	
589	-0,8080972013	000014920362868	Timp1	
831	-0,8455057328	000923690898231	Acta2;Actg2	
725	-0,9075140402	003807966348331	Cfl2	
1458	-0,9385089008	000090956596542	Htra1	
648	-1,0117285557	000689001944302	Cryab	
532	-1,0724865467	000046873801065	Tcn2	
1130	-1,0799055169	000000858192677	Tnc	
350	-1,0856945614	000000269306948	Psap	
667	-1,1643714811	0,00265317724068	Man2a1	
1449	-1,192053546	000074143866288	Adamts5	
938	-1,2469632154	000000146246211	Inhba	
920	-1,3648913543	000066440074720	Cpe	
1474	-1,4557411337	000862540127963	Asah1	
550	-1,5380418995	000002629700399	Ctsl	
681	-1,6228056021	000700494506329	Grn	
456	-2,0872097866	000092161779016	Tinagl1	
645	-2,5922890841	000078255344549	Tgm2	
692	-2,6144571361	000580776236758	Des	
